# Quantitative effect of target translation on small RNA efficacy reveals a novel mode of interaction

**DOI:** 10.1093/nar/gku889

**Published:** 2014-10-07

**Authors:** Anat Lavi-Itzkovitz, Neil Peterman, Daniel Jost, Erel Levine

**Affiliations:** Department of Physics and FAS Center for Systems Biology, Harvard University, Cambridge, MA 02138, USA

## Abstract

Small regulatory RNAs (sRNAs) in bacteria regulate many important cellular activities under normal conditions and in response to stress. Many sRNAs bind to the mRNA targets at or near the 5′ untranslated region (UTR) resulting in translation inhibition and accelerated degradation. Often the sRNA-binding site is adjacent to or overlapping with the ribosomal binding site (RBS), suggesting a possible interplay between sRNA and ribosome binding. Here we combine quantitative experiments with mathematical modeling to reveal novel features of the interaction between small RNAs and the translation machinery at the 5′UTR of a target mRNA. By measuring the response of a library of reporter targets with varied RBSs, we find that increasing translation rate can lead to increased repression. Quantitative analysis of these data suggests a recruitment model, where bound ribosomes facilitate binding of the sRNA. We experimentally verified predictions of this model for the cell-to-cell variability of target expression. Our findings offer a framework for understanding sRNA silencing in the context of bacterial physiology.

## INTRODUCTION

Small regulatory RNAs (sRNAs) are important regulators of cellular activity in a wide range of organisms, from bacteria to animals (1,2). In bacteria, they regulate all aspects of the cellular physiology, including stress response, metabolism and virulence (2,3). One of the most extensively studied classes of bacterial sRNAs consists of *trans*-acting sRNAs that are expressed independently from their protein-coding targets. Many sRNAs regulate the rate of protein synthesis of their targets by specifically binding their cognate mRNAs. Binding specificity is achieved through imperfect base pairing between a short sequence in the small RNA and a partially complementary sequence in the target mRNA, known as the seed region and sRNA-binding site, respectively. Binding often leads to suppression of translation as well as degradation of the target mRNA, sometimes accompanied by co-degradation of the sRNA itself (2,4).

How features of an sRNA target are related with the efficacy of its regulation is not well understood. sRNA binding often occurs at the 5′ end of the transcript, suggesting that the location of the sRNA-binding site, and perhaps interaction with the ribosome, is important for function (5–11). However, while it was initially believed that sRNA binding should occur at or near the ribosomal binding site (RBS), it has been demonstrated more recently that binding tens of bases away from the start codon can still influence translation in *Escherichia coli* (12–14) and *Salmonella* (15–17). sRNAs that primarily regulate the stability of target mRNA can do so from sites away from the ribosome binding site, including the coding region and the 3′ untranslated region (UTR) (18).

The rate of translation initiation is governed by the abundance of free ribosomes in the cell and is tied directly to the physiology of the cell and to its growth rate (19). The translation initiation efficiency of a given target is strongly dependent on the sequence and structure of its 5′UTR, and in particular on the similarity of the RBS sequence to a consensus sequence, known as the Shine–Dalgarno sequence (20). Any effect of an sRNA on the initiation step of translation may have different quantitative properties for target mRNAs with markedly different RBS sequences and contexts, or even the same target mRNA under different physiological conditions. Understanding the effect of translation efficiency on efficacy of small RNA regulation is therefore important for studying the evolutionary role of sRNA regulation under different physiological conditions, its impact on the evolution and co-evolution of the 5′UTRome, as well as for the design of synthetic small RNA–target pairs.

The interaction between sRNAs and the ribosome can affect the efficacy of sRNA regulation in a number of different ways. sRNA binding has been suggested to compete with ribosome binding, a model similar to the one proposed for normal mRNA degradation (21). In this model, efficient translation renders the sRNA ineffective. Alternatively, the positive effect of translation on mRNA stability in bacteria (22), combined with the predicted efficiency of sRNA regulation for stable targets (23), could lead to positive correlation between the translation rate of a gene and its susceptibility to sRNA regulation. In addition, interactions between ribosomes and Hfq, an RNA chaperone involved in RNA regulation, may add to the effect of translation on sRNA regulation. We therefore hypothesized that a quantitative study of the interactions between translation and sRNA regulation may lead to new insights into the mechanism of gene regulation by small RNAs.

Here we develop a quantitative coarse-grained model for the interaction between small RNAs and the translation machinery at the 5′UTR of an mRNA target. We show how competing models produce very different quantitative predictions. To discern between these models, we study libraries of reporter targets that differ in their RBSs, which span two orders of magnitude of translational efficiency. Our results suggest a novel indirect interaction between the translational machinery and the small RNA complex, in which translation effectively recruits the sRNA to the regulatory site. This proposed mechanism predicts the existence of distinctive signatures in the fluctuation spectrum of an sRNA–target pair, which we verify experimentally. We discuss the implications of our results on the understanding of sRNA silencing mechanisms and on modeling post-transcriptional regulatory circuits in the context of bacterial physiology.

## MATERIALS AND METHODS

### Model

**Figure 1.**
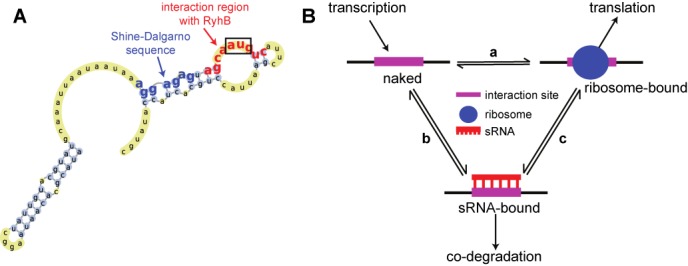
Three-state model for the interaction between the sRNA, mRNA and ribosomes. (**A**) Secondary structure of the 5′ end of *E. coli* sodB mRNA (7,30). Predicted RBS is in bold blue, the interaction region with the sRNA RyhB is in bold red. The start codon is boxed. (**B**) Three-state model for the mRNA interaction region. Ribosome (a) or sRNA (b) may bind to the transcribed naked mRNAs, leading, respectively, to translation of the mRNA or co-degradation of the sRNA–mRNA complex. Bound ribosomes and the sRNA complex may interact, directly or indirectly (c).

In our model (Figure 1B and Supplementary Figure S1) we consider three possible states for the mRNA, depending on the occupation of the interacting region (ribosome-bound, sRNA-bound or naked). Assuming a fast equilibration of the sRNA–mRNA complex, that binding–unbinding of the ribosome to the RBS is rapid and that the reservoir of free ribosomes remains large, the kinetics of the average number of mRNA *m*, of sRNA *s* and of protein *p* follow the set of mass-action equations (see the Supplementary text)

(1a) \begin{equation*} \frac{{dm}}{{dt}} = \alpha _m - \beta _m m - ks \cdot m \end{equation*}

(1b) \begin{equation*} \frac{{ds}}{{dt}} = \alpha _s - \beta _s s - ks \cdot m \end{equation*}

(1c) \begin{equation*} \frac{{dp}}{{dt}} = \gamma m - \beta _p p \end{equation*}

with *α_m_* and *α_s_* being the transcription rate of, respectively, the mRNA and the sRNA, *β_m_*, *β_s_* and *β_p_* the degradation rate of the mRNA, the sRNA and of the protein, *k* the interaction rate between the sRNA and the mRNA and *γ* the translation rate of the mRNA. *β_m_*, *k* and *γ* are coarse-grained parameters accounting for the presence of ribosomes at the interaction site:

(2a) \begin{equation*} \beta _m = \beta _{m0} \left( {\frac{{1 + wx}}{{1 + x}}} \right) \end{equation*}

(2b) \begin{equation*} k = k_0 \frac{{(1 + xy)}}{{(1 + x)(1 + z + xyz)}} \end{equation*}

(2c) \begin{equation*} \gamma = \gamma _0 \left( {\frac{x}{{1 + x}}} \right), \end{equation*}

where *x* represents the binding affinity of the ribosome and the RBS, *w* is the ratio between the degradation rates of the ribosome-bound state and the naked state, *y* is the ratio between the sRNA–mRNA interaction rates in the presence and in the absence of a ribosome at the binding site and *z* is the ratio between the dissociation rate of the sRNA–mRNA complex and the degradation rate of the complex. These parameters themselves encompass more microscopic underlying processes such as structural rearrangements of the molecules, interactions with the RNA chaperone Hfq (7) and recruitment or activation of RNases (22) (see the Supplementary text). The mean steady-state levels of *m, s* and *p* are obtained by setting the temporal derivatives on the left-hand sides of Equations (1a)–(1c) to zero and solving the corresponding set of nonlinear equations.

Stochastic properties of the mass-action system (Equations (1a)–(1c)) are captured by a chemical master equation that describes the dynamics of the joint probability distribution of the system. In the limit of weak noise, fluctuations are given by solving the linear fluctuation–dissipation relation (24)

(3) \begin{equation*} JC + CJ^T + N = 0 \end{equation*}

with *C* being the covariance matrix of the system, *J* the Jacobian of the set of mass-action Equations (1a)–(1c) and *N* is the so-called diffusion matrix and captures the different sources of noise. Complete details of the derivation and analysis of the model are given in the Supplementary text.

**Figure 2.**
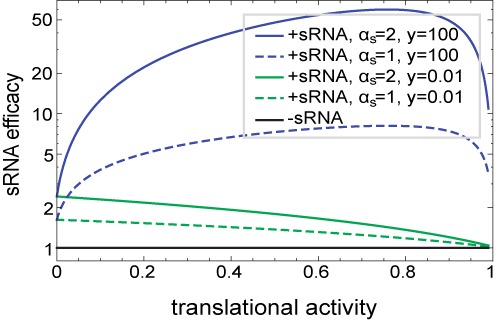
Competing models for sRNA–ribosome interactions yield qualitatively different predictions. Model predictions of the sRNA efficacy (fold-repression in gene expression) as a function of the translational activity for the competition (*y* = 0.01, green lines) and the recruitment modes (*y* = 100, blue lines) in the crossover (*α_s_*/*α_m_* = 1, dashed lines) or silenced (*α_s_*/*α_m_* = 2, full lines) regimes. Fixed parameters are (in min^−1^) *k*_0_ = 0.04, *α_m_* = 1, *β_s_* = 0.1, *β_m_*_0_ = 0.4, *w* = 1, *z* = 0.001.

Model predictions plotted in Figures 2 and 5A were calculated using typical parameter values of bacterial sRNA pathways (27). Fixed parameters are (in min^−1^) $\lambda _0 = 1,\gamma _0 = 1,\alpha _m = 1,\beta _s = 0.1,\beta _{m0} = 0.42,\beta _p = 1/60,w = 1,z = 0.001$.

**Figure 3.**
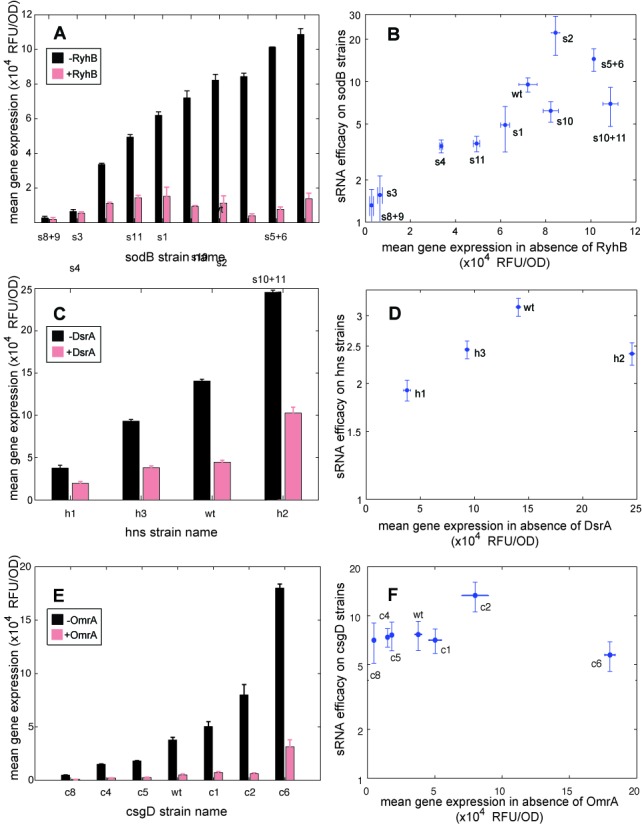
Efficient translation increases the efficacy of gene silencing. (**A, C, E**) Gene expression (measured by GFP fluorescence per OD_600_) for (A) 10 *sodB*, (C) four *hns* and (E) seven *csgD* variants with and without induction of RyhB, DsrA (aTc = 0 or 8 ng/ml, IPTG = 0.5 mM in both), or OmrA (IPTG = 0 or 1.0 mM, aTc = 10 ng/ml in both). (**B, D, F**) sRNA efficacy (defined as the ratio of target expression in the presence and absence of the sRNA) with respect to each variant, measured for (B) RyhB-*sodB*, (D) DsrA-*hns* and (F) OmrA-*csgD*, plotted against expression in the absence of the sRNA.

**Figure 4.**
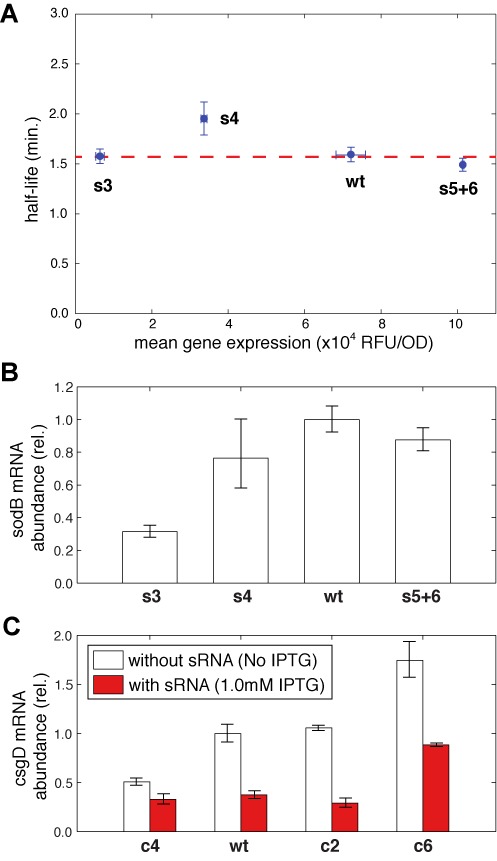
RNA stability is not strongly affected by translation rate. (**A**) Half-life of sodB mRNA for the wild-type and three RBS mutants, s3, s4 and s5+6, measured by RT-PCR, plotted against the mean gene expression in the absence of sRNA. The dashed line represents the average half-life, 1.6 ± 0.1 min. (**B**) Steady-state mRNA abundance, measured by RT-PCR in the absence of RyhB for the same *sodB* variants. (**C**) Steady-state mRNA abundance of for *csgD* and RBS mutants, c2, c4 and c6, with and without induction of OmrA by IPTG.

**Figure 5.**
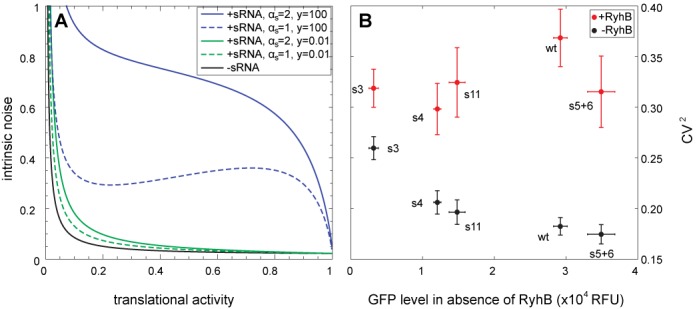
Translational recruitment leads to anomalous fluctuations. (**A**) Model predictions for the intrinsic noise *η* as a function of the translational activity for the competition (*y* = 0.01, green lines) and recruitment modes (*y* = 100, blue lines) in the crossover (*α_s_*/*α_m_* = 1, dashed lines) or silenced (*α_s_*/*α_m_* = 2, full lines) regimes. The black line represents the noise in the absence of sRNA. Fixed parameters are (in min^−1^) *λ*_0_ = 1, *γ*_0_ = 1, *α_m_* = 1, *β_s_* = 0.1, *β_m_*_0_ = 0.42, *β_p_* = 1/60, *w* = 1, *z* = 0.001. (**B**) Coefficient of variation squared (CV^2^) computed as the ratio between the variance and the mean squared of the GFP distribution obtained by flow cytometry in the absence (black) or in the presence (red) of RyhB, for five different *sodB* variants, plotted against the mean GFP level in the absence of RyhB.

### Strains and plasmids

All experiments were performed with BW-RI cells derived from *E. coli* K-12 BW25113 (25), with the transfer of the *sp^r^-lacI-tetR* cassette from DH5α-ZI cells (26) by phage P1 transduction. This cassette provides the constitutive expression of *lacI* and *tetR* genes (26). For experiments on the RyhB–*sodB* interaction, *ryhB* was additionally deleted from BW-RI (25). These strains were then transformed by the following plasmids.

pZE12S (P_Llac-O1_:*crsodB-gfpmut3b*) and pZA31R (P_Ltet-O1_:*ryhB*) plasmids were described elsewhere (27). *gfpmut3b* in pZE12S flanked by KpnI-XbaI sites was replaced by superfolder-gfp (*sfgfp*) (28) to yield pZE12SF. pZE12SF was used as a template in site-directed mutagenesis with the QuikChange II Site-Directed Mutagenesis Kit (Agilent) (see Supplementary Table S1). The RBS predictor tool (29) was used to design different sequences with a wide range of ribosome binding strengths for *crsodB* (see Supplementary Table S1 and Supplementary Figure S8). Mutations were chosen only if they have little or no effect on the secondary structure and on the sRNA-binding affinity as tested using the ViennaRNA package (30–32) (Supplementary Table S2 and Supplementary Figure S2). In some cases, a complementary mutation was created in order to keep the secondary structure (s5+6 and s8+9, marked as * in Supplementary Table S1). Mutations were verified by sequencing.

P_Llac-O1_-*crsodB-sfgfp* wild-type and all the mutant sequence variants were cloned from pZE12SF and ligated into the XhoI-XbaI sites of pAS04 (a gift from P. Cluzel), a low copy number plasmid with pSC101* *ori* (26) to yield the pAS05 library. pAS05 library therefore contains the pSC101* *ori*, the P_Llac-O1_ promoter (26) and *crsodB* (wild type or one of the mutants) fused to the coding sequence of *sfgfp*.

For experiments involving *hns* and DsrA, the 5′UTR and first 11 codons of *sodB*, between the EcoRI-KpnI sites on pAS05, were replaced with 36 bases in the 5′UTR and the first 28 codons from pHns::gfp (a gift from J. Vogel, described in (33)) by subcloning in order to produce pAS07. For experiments on *csgD* and OmrA, the sRNA expression plasmid pOmrA and target-reporter pCsgD::GFP were constructed as described in (34). For pOmrA and pCsgD::GFP, the sRNA is induced by isopropyl β-D-1-thiogalactopyranoside (IPTG) and the target-reporter by aTc.

Target-reporter plasmids for *hns* and *csgD* with several different RBS sequences were designed as above (Supplementary Figure S8B and C). Variants for *hns* were synthesized with QuikChange II Site-Directed Mutagenesis Kit (Agilent) using pAS07 as a template and primers listed in Supplementary Table S1. For *csgD*, variants were synthesized with Q5 Site-Directed Mutagenesis Kit (NEB) using pCsgD::GFP as a template (see Supplementary Table S1 for primers). Mutations were verified by sequencing.

The *ryhB* gene in pZA31R was replaced with a random fragment to yield the control vector pZA31-RF. pZA31D was cloned by replacing *ryhB* between NdeI-BamHI sites in pZA31R with *dsrA* from pBRdsrA (a gift from M. Belfort, described in (35)).

### Medium, growth, measurements

BW-RI strains each harboring a target plasmid (pAS05, pAS07 or pCsgD::GFP) wild type or one of the mutant variants and an sRNA plasmid (pZA31R, pZA31-RF,pZA31D or pOmrA) were grown in M63 minimal media with 0.5% glucose, 0.1% Casamino Acids and standard concentrations of the appropriate antibiotics. The overnight cultures were washed and diluted 1/166 into fresh M63 containing the appropriate antibiotics and incubated with shaking at 37°C to recover. At 0.1<OD_600_<0.2, the cultures were diluted to OD_600_ = 0.005 with varying amounts of the inducers (aTc, IPTG) in a 48-well plate at 1 ml per well. After 3 h (when OD_600_ came close to 0.2), cultures were diluted again to OD_600_ = 0.005 into a new 48-well plate with the same concentration of inducers. After additional 2 h of growth in the incubator, the plate was placed in a FLUOstar OPTIMA (BMG Labtech) with shaking at 37°C, where absorbance (595 nm) and green fluorescent protein (GFP) fluorescence (Em 485, Ex 520) measurements were taken every 10 min for up to 2 h.

### RNA stability assay

This protocol is based on the RNA turnover protocol from (36). Overnight cultures of BW-RI Δ*ryhB* strains each harboring the plasmids pAS05 wild type or the mutant strains and pZA31-RF were used to inoculate M63 medium with 0.5% glucose, 0.1% Casamino Acids and standard concentrations of the appropriate antibiotics and 1-mM IPTG to an initial OD_600_ of 0.01 and grown in flasks in a bath shaker at 37°C. OD_600_ was monitored periodically. When OD_600_ of these cultures reached 0.2–0.5, 500-μg/ml Rifampicin (Sigma-Aldrich) was added to each flask and 120 s later the first sample of 0.2 ml was collected (this was defined as time point 1 min), followed by four more collection time points. Additionally, a no Rifampicin control was collected for wild-type *sodB* strain. Samples were added immediately into 0.4 ml of RNA protect bacteria reagent (Qiagen) and vortexed. After 5 min at room temperature samples were centrifuged for 1 min at 10 000xg and the pellet was flash-frozen. RNA extraction was done simultaneously for all samples from the same strain using the RNeasy Mini Kit (Qiagen). RNase-Free DNase (Qiagen) was added to the columns during RNA extraction for 15 min. cDNA was prepared from 0.2–1-μg RNA of each sample using qScript cDNA SuperMix (Quanta Biosciences). Dilutions of the resulting samples were then used as template in reverse transcriptase polymerase chain reaction (RT-PCR) using PerfeCTa SYBR Green SuperMix (Quanta Biosciences) in Mastercycler RealPlex2 (Eppendorf). For RT-PCR with the internal control 16S, samples were diluted 1/1000. RT-PCR was performed in duplicates, and a no-template control was included in each experiment. Primers used: 16SF CTCCTACGGGAGGCAGCAG; 16SR GTATTACCGCGGCGCTG; sfgfpF GATCCGTTCAACTAGCAGAC; sfgfpR ACAGGTAATGGTTGTCTGGT.

For steady-state mRNA abundance measurements, overnight cultures were washed and diluted 200-fold in fresh M63 medium with 0.5% glucose, 0.1% Casamino Acids and antibiotics. After the cultures reached OD_600_ 0.1–0.2 they were diluted to OD_600_ = 0.001 in 48-well plates, 1-ml media with appropriate inducers (1.0-mM IPTG for *sodB* and 10-ng/ml aTc for *csgD*), and incubated on a plate shaker at 37°C. After 4–5 h (OD_600_ measured between 0.1 and 0.2), 0.3 ml was transferred to 0.6-ml RNAprotect Bacteria Reagent (Qiagen), and then vortexed, incubated, pelleted and frozen as above. RNA extraction was done with RNeasy Mini Kit (Qiagen), and cDNA synthesis was carried out with 0.3-μg RNA using the SuperScript III for RT-PCR (Invitrogen). RT-PCR was performed as above using SYBR FAST qPCR Master Mix (Kapa Biosystems).

### Flow cytometry

To measure noise properties, BW-RI Δ*ryhB* strains each harboring the target plasmid pAS05 wild type or one of the sequence variants and pZA31R plasmid were grown in M63 minimal media with 0.5% glucose, 0.1% Casamino Acids and standard concentrations of the appropriate antibiotics. The overnight cultures were washed and diluted 1/166 into fresh M63 containing the appropriate antibiotics and incubated with shaking at 37°C to recover. OD_600_ was measured periodically. At 0.1<OD_600_<0.2, the cultures were diluted to OD_600_ = 0.005 with varying amounts of the inducers (aTc, IPTG) in a 48-well plate at 1 ml per well and grown in a 37°C incubator with constant shaking. After 3 h (when OD_600_ approaches 0.2), cultures were diluted again to OD_600_ = 0.005 into a new 48-well plate with the same concentration of inducers. At 0.1<OD_600_<0.2, 5 μl from each well was transferred into 1 ml of phosphate buffer solution in a 96-well plate.

GFP fluorescence was measured using BD LSRFortessa cell analyzer with the high-throughput sampler unit, with a 505-nm excitation laser and a 530/30 emission filter at a low flow rate. Photomultiplier tube voltage for fluorescein isothiocyanate was set to 600 V. A lower threshold was set for side scatter. Forward scatter, side scatter and fluorescence values were collected for 50 000 cells. A no-IPTG control (wild-type strain) was included in each experiment.

### Data analysis

#### Mean gene expression

To estimate target expression in a given strain we performed fluorescence measurements in multiple (3-5) repeats, with three technical replicates (adjacent wells on a microplate) in every repeat. ‘GFP per cell’ in a particular well was defined as the slope of the GFP versus OD curve in its linear part (that is, during exponential growth and within the linear detection range of the plate reader, see Supplementary Figure S3). For every biological repeat, we averaged the three replicates, and corrected it by subtracting from this average an estimate for auto-fluorescence. The latter was obtained by measuring ‘GFP per cell’ in un-induced cultures. Finally, we define ‘gene expression’ (measured in relative fluorescence units, RFU/OD) for a particular strain as the average of the corrected ‘GFP per cell’ over the different biological repeats. In summary, the mean gene expression *g^s^* and its corresponding standard error $\sigma _g^s$ were estimated as $g^s = n_s^{ - 1} \sum\nolimits_r {g_r^s }$ and $\sigma _g^s = n_s^{ - 1} \sqrt {\sum\nolimits_r {(g_r^s - g^s )^2 + \sigma _{g_r^s }^2 } }$, with *n_s_* being the number of repeats for strain *s*, $g_r^s$ the normalized mean slope measured at repeat *r* and $\sigma _{g_r^s }$ the corresponding standard error.

#### Half-life

To estimate mRNA half-life from RT-PCR data for a given experiment, we averaged the *C*_q_ values of three replicates at each time point and used the efficiency corrected method (ΔΔ*C*_q_, (37)) to determine the number of mRNA molecules in each sample relative to internal control. The corresponding time evolution of the mRNA relative amounts (Supplementary Figure S4) was fitted by the function $f(t) = f_0 2^{ - t/\tau _{1/2} }$, with $\tau _{1/2}$ being the half-life and *f*_0_ a constant. For each strain, different repeats were averaged to obtain the mean half-life and the corresponding standard error. Data point at *t* = 7 min was not included in the fit for strain s3 due to low signal-to-noise.

#### Noise

To obtain mean gene expressions and noise levels for the different strains from flow cytometry experiments, we extract the mean and the variance of the distribution of GFP level (in RFU) given by the flow cytometer (Supplementary Figure S5). For each repeat, replicates were averaged to obtain the mean total GFP level and the corresponding variance. From each GFP level and variance, we subtracted the contribution of auto-fluorescence which was measured independently using an un-induced wild-type *sodB* strain.

From these corrected values, for each strain *s* and each repeat *r*, we estimated the noise level $\eta _r^s$ defined as the coefficient of variation squared (i.e. the ratio between the variance and the mean squared). Finally, for each strain, repeats were averaged to obtain the mean noise level (y-axis of Figure 5B) and its standard error, $\eta ^s = n_s^{ - 1} \sum\nolimits_r {\eta _r^s } ,\sigma _\eta ^s = n_s^{ - 1} \sqrt {\sum\nolimits_r {(\eta _r^s - \eta ^s )^2 + \sigma _{\eta _r^s }^2 } }$ with *n_s_* being the number of repeats for strain *s* and $\sigma _{\eta _r^s }$ the standard error of the noise for repeat *r* computed over the replicates. The mean GFP level (horizontal axis of Figure 5B) and its standard error were computed in an analogous fashion.

### Bioinformatics

To study the conservation of sRNA-binding site locations, we first selected a set of 600 genes that are highly conserved across Enterobacteriaceae (FASTA alignment score higher than 1000 in all considered species, *E. coli*, *Salmonella enterica*, *Enterobacter cloacae*, *Klebsiella pneumoniae*, *Pantoea ananatis*, *Shigella flexneri*, *Dickeya dadantii*, *Yersinia pestis*, *Edwardsiella ictaluri*, *Serratia plymuthica*). In particular, nine genes in this set (*OmpR*, *luxS*, *phoP*, *ftsZ*, *ptsG*, *fur*, *hns*, *sdhA* and *tpx*) are experimentally validated sRNA targets in *E. coli* (25). For this subset, the bioinformatic tool RNAup (32) was used to identify the sRNA-binding site in all other species, its distance from the start codon was estimated and the variance in this distance across all species was calculated. As a background model we considered the other 591 genes of the original set. For each gene, we assigned 10 ‘mock-sRNAs’ that have ‘seeds’ complementary to a random 10-nt sequence in the 5′UTR of that gene in *E. coli*. Again we used RNAup to identify the ‘binding site’ of each mock-sRNA in the corresponding gene of all other species and calculated the variance in their distance from the start codon.

## RESULTS

### Competing models for translation under sRNA regulation

In bacteria, negative sRNA regulation can be described by a model that includes the synthesis of all RNA species, interaction of the sRNA with its targets and the consequent degradation of the sRNA–mRNA complex (27,38–42). This model can be translated into a simple mathematical framework (27). Previous studies of this model focused on the effect the sRNA confers on the mRNA abundance of its target, assuming a fixed translation rate.

In many cases, however, the binding site for the sRNA is adjacent to the RBS and the start codon in the target mRNA (Figure 1A). About 85% of the experimentally known binding sites of sRNAs repressors are located in a region between 40 nt upstream and 20 nt downstream of the start codon (Supplementary Figure S6A), a region that likely represents the area of influence of a ribosome bound to the RBS. Such proximity suggests that ribosomes and sRNAs can affect the binding efficiency of each other, for example by competing for the same binding site, by modulating the structure of the RNA scaffold or by cooperatively assisting in binding. If this proximity is biologically functional, we expect the position of an sRNA-binding site to be under selection. To test this hypothesis, we performed a simple bioinformatic analysis for nine known sRNA–mRNA pairs in *E. coli* (see the Materials and Methods section). In eight out of the nine, the binding sites are located within 20 nucleotides from the start codon (Supplementary Figure S6A), and this location is significantly more conserved than those of arbitrary segments of similar size in the 5′UTR of conserved genes (K-S test, *P*-value <10^−3^; Supplementary Figure S6B). Interestingly, among the investigated pairs the one whose location is the least conserved (the binding site of RyhB on the mRNA of *fur*) is also the one that is the farthest from the start codon (red square in Supplementary Figure S6).

We therefore sought to develop a model for sRNA regulation that accounts for the presence of a ribosome at the interaction site and to explore the effect of ribosome binding on sRNA regulation. We considered three possible states of the mRNA target, depending on the occupation of the relevant 5′ interaction region: ribosome-bound, sRNA-bound or naked. Transitions between these different states are depicted in Figure 1B. In particular, we allow the presence of a ribosome at the interaction site to affect two key processes controlling the strength of the regulation (29): the sRNA–mRNA interaction rate and the degradation rate of the mRNA (see the Supplementary text). The coarse-grained dynamics of the average numbers of mRNA, sRNA and protein is then captured by a set of mass-action equations (Equations (1a)–(1c) in the Materials and Methods section).

Depending on the production rate of the sRNA, the mean expression level of the protein exhibits three different regimes (Supplementary Figure S7A) (27): (i) an expressed regime (occurring under conditions in which the transcription rate of the target gene *α_m_* is significantly higher than the transcription rate *α_s_* of the sRNA gene, *α_m_>>α_s_*) where the presence of the sRNA only weakly affects the mRNA pool and the protein is normally expressed; (ii) a silenced regime (*α_m_<<α_s_*) where most of the mRNAs are targeted by the large pool of sRNA and the expression of the protein is very low; and (iii) a crossover regime (*α_m_* ≈ *α_s_*) where the production rates of the sRNA and the mRNA are similar, allowing for fine-tuning of the gene expression. The ‘efficacy’ of sRNA regulation—quantified through the ratio between target expression in the presence and absence of the sRNA–is controlled by the ‘leakage rate’ *λ* that compares the time scales of the different RNA turnover mechanism (see Supplementary text). In particular, a low *λ* value leads to strong repression and sharp linear-threshold response (Supplementary Figure S7A).

To account for the effect of translation on the efficacy of sRNA repression we define two parameters. The first, denoted by *w*, is defined as the ratio between degradation rates of mRNA molecules that are bound or unbound to an initiating ribosome. In particular, *w* < 1 signifies a ‘protection’ conferred on the mRNA by bound ribosomes. The second parameter, denoted by *y*, is defined as the ratio of sRNA–mRNA-binding rates in these two states and accounts for the interactions (either direct or indirect) of the ribosome and the sRNA at the 5′UTR. In particular, *y* < 1 connotes competition for binding between the ribosome and the sRNA, whereas *y* > 1 suggests that ribosomes perhaps recruit sRNA.

The effect of translation on the sRNA efficacy turns out to depend strongly on the ratio between these two parameters, which defines two distinct cases. First, the case *y*/*w* < 1 accounts both for the competition model, where sRNA and ribosome compete for the same binding site, and for the less likely scenario in which ribosome binding promotes mRNA degradation. Either way, our model predicts that in this case the sRNA efficacy is a monotonically decreasing function of the ribosome binding affinity (Figure 2, green lines).

The second case, corresponding to *y*/*w* > 1, encompasses mRNA protection by translating ribosomes, as well as the possibility of sRNA recruitment by ribosomes. The latter may occur either by direct interactions between ribosomes and sRNAs or proteins involved in sRNA regulation such as Hfq (7), or, for example, by allosteric changes to the mRNA that favor sRNA binding (see the Supplementary text). The main effect of ribosome binding in this model is to increase the efficacy of sRNA repression (Figure 2, blue lines). However, at high ribosomal binding affinities the ribosome outcompetes even resident sRNAs and translation once again inhibits sRNA repression. Together, the model predicts a non-monotonous dependence of repression efficacy on ribosome binding strength: increasing at low affinities and decreasing at high affinities, with a maximum at the place where the two sRNA-binding channels are somewhat balanced.

### Efficient translation increases the efficacy of gene silencing

Given the clear predicted quantitative signatures of the different possible effects of translation, we hypothesized that a simple quantitative experiment could shed light on the interactions between the translation machinery and small RNAs. We focused mainly on one well-characterized target–sRNA pair from *E. coli*, in which we experimentally modified the ribosome binding strength and assessed the effect on target regulation.

As a model system we chose RyhB, a small RNA involved in regulation of iron homeostasis in *E. coli*, and *sodB*, which encodes a superoxide dismutase (43–45). Binding of RyhB to the sodB mRNA, facilitated by the RNA chaperone Hfq (7,44), leads to co-degradation of the two RNA molecules (45). We used a synthetic target gene consisting of the 5′ control region and the first 11 codons of *sodB* (*crsodB*) (27) translationally fused the reporter superfolder-gfp (*sfgfp*, (28)). The target gene, *crsodB-sfgfp*, was placed on a low-copy number plasmid pAS05. The *ryhB* gene was placed on a different plasmid, pZA31, driven by the strong synthetic PLtet-01 promoter (26) inducible by anhydrotetracycline (aTc). This construct allowed us to control the transcription rate of the sRNA. We next used site-directed mutagenesis of one or two nucleotides to create nine different variants of *crsodB-sfgfp* with different Shine–Dalgarno sequences (Supplementary Figure S8A) (29,46). Each variant was co-transformed into a Δ*ryhB* background along with the plasmid containing *ryhB* (or an empty vector) to generate a library of 10 strains. In choosing the sequence variants for this study we took great care to select variants predicted to conserve the same secondary structure, Hfq-binding site and sRNA-binding affinity as the wild-type sequence (Supplementary Table S2 and Supplementary Figure S2) in order to preserve the pairing properties with RyhB while altering the translation rate.

To characterize the relative ribosomal binding strength of the variants in our library we compared GFP expression without sRNA induction (0.5-mM IPTG, no aTc added) in a microplate reader. The different strains in our library demonstrated a wide range of GFP expression levels (Figure 3A, black bars), which correlated with their predicted ribosome binding strength (Supplementary Figure S9A). To measure the effect of the ribosome binding strength on sRNA efficacy, we repeated our measurements with induction of RyhB (IPTG = 0.5 mM, aTc = 8 ng/ml) (Figure 3A, red bars). In most strains, the presence of RyhB reduced GFP expression, as expected from the repression of *sodB* by RyhB.

We found that the efficacy of RyhB repression (measured as the ratio of GFP fluorescence per cell in the presence and absence of the sRNA) tends to rise with increasing reporter translation efficiency and eventually decreases (Figure 3B). Maximal sRNA efficacy occurred at high translation rates. Interestingly, we found that the wild-type sequence corresponds to one of the most efficiently regulated variants. Thus, in the language of our model, the dynamics of the RyhB–*sodB* pair belong to the case *y*/*w* > 1.

While several molecular mechanisms behind this positive effect of translation on sRNA repression are possible, we hypothesized that the mechanism at play involves local interactions between the translation machinery and the small RNA (mediated, perhaps, by auxiliary molecules like Hfq). If this is the case, mRNA targets with different arrangements of adjacent RBSs and sRNA-binding sites may also show a similar effect. To test this possibility, we considered a different well-studied pair, the *hns* gene and its sRNA repressor DsrA. This small RNA is expressed at low temperatures to activate expression of the sigma factor *σ^s^* and repress expression of the nucleoid structuring protein H-NS (35,47). Unlike the RyhB–*sodB* pair, where the sRNA-binding site occurs between the RBS and the start codon, binding of DsrA to the hns mRNA occurs immediately downstream of the start codon (Supplementary Figure S10A).

Using a similar approach to the one described above we constructed a library of *crhns-sfgfp* target reporters with four different RBSs (Supplementary Figure S8B), and co-transformed each along with a DsrA expressing plasmid (or an empty vector). We measured the changes in translation efficiency and the corresponding efficacy of repression for these strains as above (Figure 3C). Once again, we find that increasing ribosome binding efficiency first increases and then decreases the sRNA efficacy (Figure 3D). Interestingly the wild-type RBS exhibits the highest observed sRNA efficacy, as seen above for *sodB*. Thus, the effect of translation on DsrA-*hns* regulation is similar to the one observed for the RyhB–*sodB* pair, corresponding to *y*/*w* > 1.

In contrast, the hypothesis that a positive effect of translation on sRNA regulation requires proximity between the RBS and the sRNA-binding site suggests that such an interaction would not be observed in mRNA targets where the two sites are distant and structurally separated. As noted above, this is not typically the case (Supplementary Figure S6A). Yet one such example is the gene *csgD*, a transcriptional activator of the curli genes in *E. coli*, which is repressed by the sRNAs OmrA and OmrB (48). Base pairing between these sRNAs and *csgD* occurs at the 5′UTR of csgD, far upstream of the RBS and in a different stem-loop structure (Supplementary Figure S10B).

To test the hypothesis that sRNA regulation of *csgD* is independent of translation efficiency we generated a 6-variant RBS library of *csgD* target reporters that spans a 20-fold range of translation levels (Supplementary Figure S8C) and tested the efficacy of OmrA in repressing these variants (Figure 3E). Despite the wide range of translation rates, the fold-change in target expression was indistinguishable for almost all targets (Figure 3F), in support of our hypothesis.

Taken together, our results support a model where a positive effect of translation on sRNA efficacy may occur for targets where the sRNA-binding site is situated near the RBS. In the two such cases we characterized this effect is positive (*y*/*w* > 1), though we cannot exclude the possibility that in other pairs it is negative.

### Translational activity promotes sRNA binding

In our mathematical model, ribosome binding site strength may affect the efficiency of sRNA regulation (via the leakage rate *λ*) by two independent mechanisms, either by modulating the sRNA–mRNA interaction (via *k* and *y*) or by affecting the mRNA degradation rate (via *β_m_* and *w*).

To explore which of these mechanisms contributes to the effect observed in the previous section, we measured the mRNA degradation rates of the different *sodB-gfp* variants by quantitative PCR (qPCR) measurements following a rifampicin treatment (36) in the absence of RyhB. We focused on four strains (s3, s4, wild type and s5+6), spanning a wide range of translation efficiencies (Figure 3A).

We found that RBS occupancy does not significantly change the mRNA stability of sodB, as all variants showed very similar half-lives with an average value around 1.6 min (Figure 4A and Supplementary Figure S4). In support of this finding, we found that the mRNA steady-state abundance of these four variants (measured by qPCR) is indistinguishable for three of the variants, and is <2-fold different than the fourth (Figure 4B). Moreover, the linear correlations between measured GFP fluorescence from these variants and the computational predictions based on ribosome affinity (Supplementary Figure S9) suggest little effect on mRNA level. These results are consistent with setting *w* ≈ 1 in our model for RyhB-*sodB*. Since our results for this pair are consistent with *y/w>*>1 we conclude that *y* > 1, namely the sRNA binds more efficiently to a ribosome-bound mRNA than to a naked one. Thus, the positive effect of translation on RyhB efficacy is mainly due to sRNA recruitment by the translation process rather than by stabilization of the mRNA.

It is interesting to note that *csgD*, whose translation rate has no effect on *omrA* efficacy, is also at most marginally stabilized by translation (Figure 4C). This finding supports the idea that translation has no effect on sRNA binding to this molecule. We note that the opposite is probably not true, since the effect of the sRNA on target expression (≳7-fold) is only partially explained by its effect on mRNA abundance (≲2-fold; Figure 4C) as reported previously (48).

### Translational recruitment leads to anomalous fluctuations

The stochastic nature of the biochemical reactions composing gene regulation pathways leads to intrinsic fluctuations around the mean signal levels (49–51). To assess the effect of ribosome–sRNA target cooperativity on target fluctuations, we augmented our mass-action formalism to account for the stochasticity of the underlying biochemical processes (see the Materials and Methods section). A canonical way to appreciate the strength of fluctuations in the system is to consider the noise *η* defined as the ratio between the variance *σ_p_^2^* of the gene expression and the square of the mean expression <*p*> (*η = σ_p_^2^*/<*p*>*^2^*).

In the absence of sRNA, the noise is given by *η =* (1 + *b*)/<*p*>, with *b* = *γ/β_m_* the so-called protein burst size representing the average number of protein produced per mRNA. As the affinity of the ribosome with the RBS increases, the mean protein level increases and the noise decreases (Figure 5A, black line).

Our model predicts that the presence of sRNA dramatically changes the noise properties (Supplementary Figure S7B). In particular, in the silenced regime sRNA regulation is very efficient to suppress intrinsic fluctuations due to the significant reduction of the effective lifetime of the mRNA. However, in the crossover regime where the transcription rates of the sRNA and the mRNA are similar, expression levels alternate between repressed and unrepressed states, leading to large highly sensitive fluctuations in the protein level (52,53). This stochasticity is enhanced by strong sRNA–mRNA interactions (low leakage rate *λ*; see Supplementary Figure S7B).

What is the effect of translational activity on the noise in the presence of sRNA? In the competition mode (*y*/*w* < 1), increasing the affinity of the ribosome with the RBS leads to noise reduction (Figure 5A, green lines), consistent in every regulated regime (expressed, crossover and silenced). In contrast, in the recruitment mode (*y*/*w* > 1), our model predicts a suppression of noise by enhanced translational activity only in the expressed and silenced regimes (Figure 5A, blue lines). In the ultra-sensitive crossover regime, on the other hand, the fluctuations may behave anomalously with the appearance of a local maximum (Figure 5A, dashed blue line) that reflects the non-monotonous relation between the leakage rate *λ* and the translational activity. The maximal noise is observed when *λ* is minimal, i.e. when the sRNA regulation is optimal in terms of fold-repression.

We hypothesized that this predicted signature could validate our previous conclusion that the sRNA is recruited by ribosome binding (Figure 3). To estimate the effect of translational efficacy on the noise in target expression, we re-examined the five sequence variants from our *crsodB-sfgfp* mutant library (wt, s3, s4, s5+6 and s11) and measured single-cell target fluorescence by flow cytometry (54). Each strain was grown with and without induction of the sRNA (aTc = 0 ng/ml and 4 ng/ml, respectively; IPTG = 0.5 mM). Noise properties were computed from the distributions of the GFP levels within the bacterial population (Supplementary Figure S5). Figure 5B shows the coefficient of variation squared of these distributions, which are equal to *η* up to an additive constant representing the contribution of extrinsic noise (50,55).

As predicted by the model, in the absence of RyhB, noise in the expression of GFP is monotonously decreasing with the translation efficiency (Figure 5B, black dots). In comparison, the presence of RyhB significantly increases the fluctuations (Figure 5B, red dots). Importantly, despite the wide range of RBS affinities and sRNA efficacies presented by our different variants, their noise levels in the presence of sRNA do not decline with translation efficiency, consistent with the recruitment model (compare with the blue dashed line in Figure 5A) but not with the competition model. In fact, our results are suggestive of the non-monotonic behavior predicted by the model near the crossover regime.

## DISCUSSION

Molecular interactions with functional impact are rarely limited to the two interacting molecules. In the cell, many co-factors facilitate and mediate such interactions and may be involved in executing its function. Biochemical characterization of the molecular interactions is often limited in the number of components and, as a result, may misrepresent the implications of physiological conditions and the cellular environment, or otherwise may be too laborious or impractical. Here we propose a top-down approach, where a quantitative study of the input–output relations *in vivo* uncovers effective interactions with functional implications.

Using a combination of mathematical modeling and quantitative experiments we revealed an unexpected effect of translational efficiency on RyhB regulation that cannot be explained by the effect of translation on mRNA stability. This observed quantitative characteristic is consistent with a model whereby sRNA is effectively recruited to its target by the presence of ribosomes at the RBS. This model predicts a distinct shape for the dependence of noise on translation efficiency, which we have verified experimentally. Thus, quantitative measurements of the dependence of the input–output relation of sRNA regulation on translation efficiency reveal a novel interaction in the mechanism of sRNA-mediated silencing.

The mechanism behind recruitment is unknown, although proximity between the RBS and the sRNA-binding site seems necessary. One possibility is a direct interaction between the ribosome and either the sRNA (56,57) or Hfq (58–60), a known RNA chaperone that binds both sRNA and target mRNA subunits and facilitates regulation (61). An alternative mechanism could involve interplay with the secondary structure of the mRNA or the structure of the sRNA–mRNA duplex. A recent high-throughput mutational study of sRNA repression suggested that changes to secondary structure of RyhB containing the seed sequence can influence its regulatory efficiency and specificity (62). Indeed, both the ribosome and sRNA-binding sites reside within a stem-loop secondary structure in the 5′UTR of sodB (7). Ribosome binding might unfold the hairpin secondary structure and thus transiently facilitate sRNA binding. In contrast, the binding site of DsrA to the hns mRNA is located immediately downstream of the start codon, away from the predicted stem-loop structure that incorporates its RBS (63,64). Precise distinction between these different mechanisms would require deeper biochemical experiments to probe the molecular details of the recruitment process.

Interestingly, our experiments revealed that the ribosome binding sites for the wild-type *sodB* and *hns* sequences are not tuned to maximal translation efficiency, but rather reside very close to the maximal efficiency for their sRNA-mediated regulation (Figure 3). Our bioinformatic analysis of the conservation of interaction site position across Enterobacteriaceae (Supplementary Figure S6) shows that locations of most of the experimentally known sRNA-mRNA sites are more conserved than expected by chance. This suggests that the position of the sRNA interaction site around the RBS is likely to be under evolutionary pressure. Thus, it is possible that evolution has tuned sequence preferences to accommodate more efficient control by the sRNA.

Our model predicts that sRNA regulation efficiency depends on the translational activity of the mRNA, and thus on the availability of free ribosomes in the cell. Consequently, it is possible that a change in physiological conditions, which affects the concentration of free ribosomes in the cell, would be accompanied by a significant change in sRNA efficacy (Supplementary Figure S11). Our model therefore suggests that the sequences of sRNA and target 5′UTR may evolve to optimize their interactions under the cellular conditions in which each sRNA functions.

We found that in the absence of the corresponding sRNAs the half-life of *sodB* mRNA does not depend significantly on translational activity (Figure 4A). Additionally *sodB* and *csgD* mRNA abundance levels in the absence of the sRNA are not greatly affected by changes in translational activity. This observation might be surprising as translation has been shown to partially protect mRNAs from degradation due to the transient covering of RNase E cleavage sites by ribosomes (22). However, Morita *et al.* observed that for *ptsG*, a negative target of the sRNA SgrS, inhibiting translation in the absence of SgrS does not significantly change the mRNA level in the cell (65). Thus, the neutral dependence of mRNA decay on the translational activity might be a property of sRNA-regulated mRNA targets.

Close inspection of the effect of translation on sRNA properties also exposes the large fluctuations that may be associated with sRNA regulation. Our model suggests that these fluctuations are enhanced by efficient repression, supported by the strong correlations we find between repression strength and intrinsic fluctuations. In this light, the high efficiency of repression in wild-type *sodB* might be viewed as problematic, as it allows significant intrinsic noise (Figure 5 and Supplementary Figure S7B) (66). However, we have recently shown that the presence of weak auxiliary targets may play a role in suppressing such fluctuations (67). For RyhB, candidates for these weak targets include *nagZ*, *metH*, *cysE*, *yciS* or *acpS* (68).

Synthetic biology is receiving rapidly growing attention as a field with potential for applications in health, food, energy and more. Progress in this field is limited by our ability to formulate predictive models for simple genetic circuits. Our finding here, which correlates the efficiency of sRNA regulation with the efficiency of target translation, should be taken into account in future attempts to involve sRNAs in designing synthetic circuits. Moreover, we believe that the link we unveiled between efficiency of silencing and the physiology of the cell is the rule rather than the exception: a key difference between genetic elements and, say, electronic gates is the fact that the function of even the simplest element is directly linked to the function of the entire system. We argue that a top-down quantitative characterization of this dependence *in vivo* may facilitate the design of genetic circuits that are more likely to be functional under the required cellular conditions.

## SUPPLEMENTARY DATA

Supplementary Data are available at NAR Online.

SUPPLEMENTARY DATA
