# Supplementary material for: Quantitative effect of target translation on small RNA efficacy reveals a novel mode of interaction
Source: Nucleic Acids Res. 2014 Oct 7;42(19):12200–11. doi: 10.1093/nar/gku889 (PMC4231754; doi:10.1093/nar/gku889)
Supplement: SUPPLEMENTARY DATA [file supp_42_19_12200__index.html]

Quantitative effect of target translation on small RNA efficacy reveals a novel mode of interaction — SUPPLEMENTARY DATA 

# Quantitative effect of target translation on small RNA efficacy reveals a novel mode of interaction

## SUPPLEMENTARY DATA

**Files in this Data Supplement:**

- SUPPLEMENTARY DATA
