# Supplementary material for: Quantitative effect of target translation on small RNA efficacy reveals a novel mode of interaction
Source: Nucleic Acids Res. 2014 Oct 7;42(19):12200–11. doi: 10.1093/nar/gku889 (PMC4231754; doi:10.1093/nar/gku889)
Supplement: SUPPLEMENTARY DATA [file supp_gku889_nar-00810-z-2014-File007.pdf]

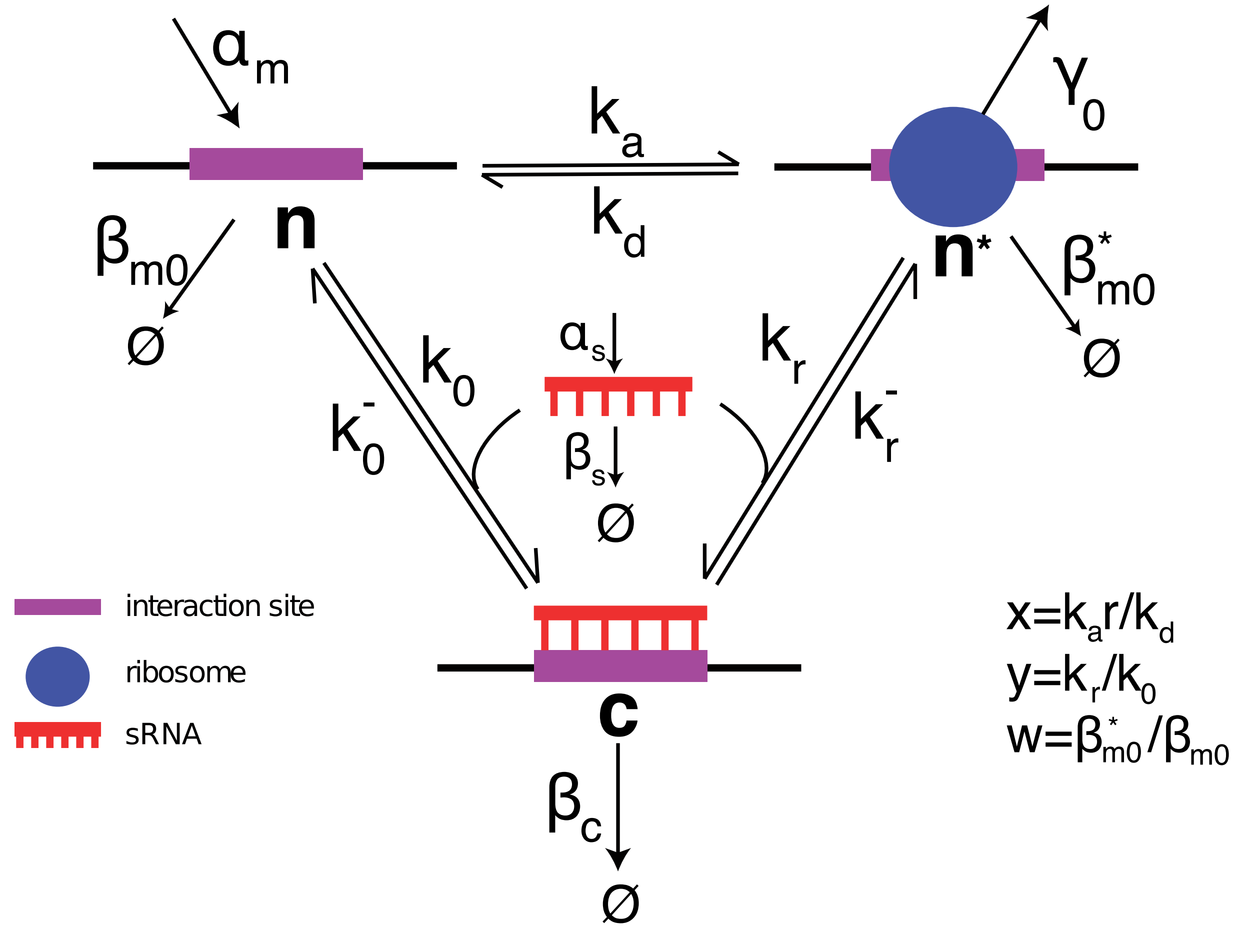

<u>Supplementary Figure S1</u>. **Three-state model for the interaction between the sRNA, mRNA and ribosomes.** Scheme of the different reactions accounted by our detailed three-state model that lead to the coarse-grain dynamics given in Eq. (1a-1c) of the main text. See Supplementary Notes for a detailed description of each reaction rate.

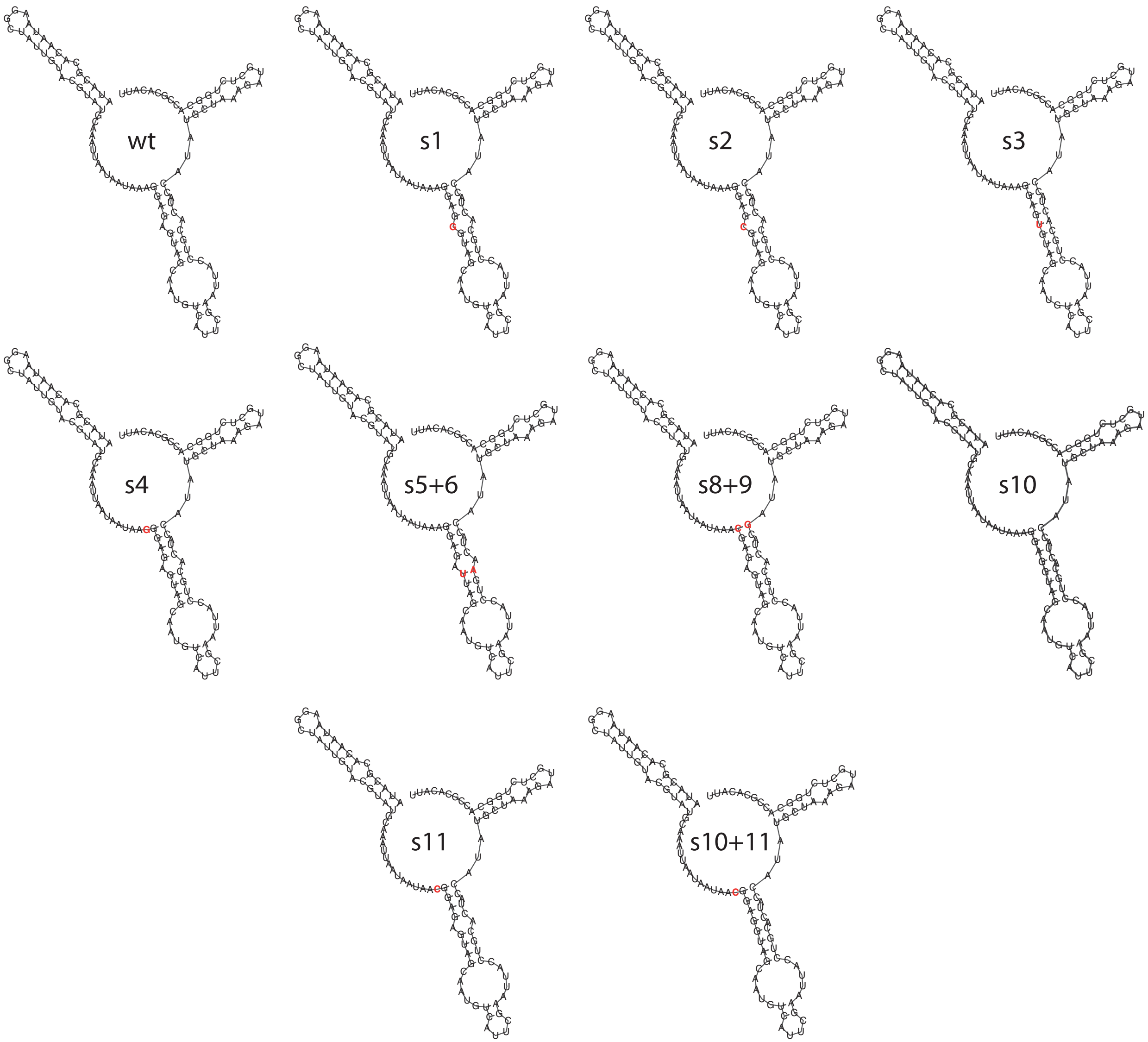

<u>Supplementary Figure S2</u>: **Minimum free energy structure for the *sodB* strains** . Minimum free energy structure as predicted by RNAfold (32).

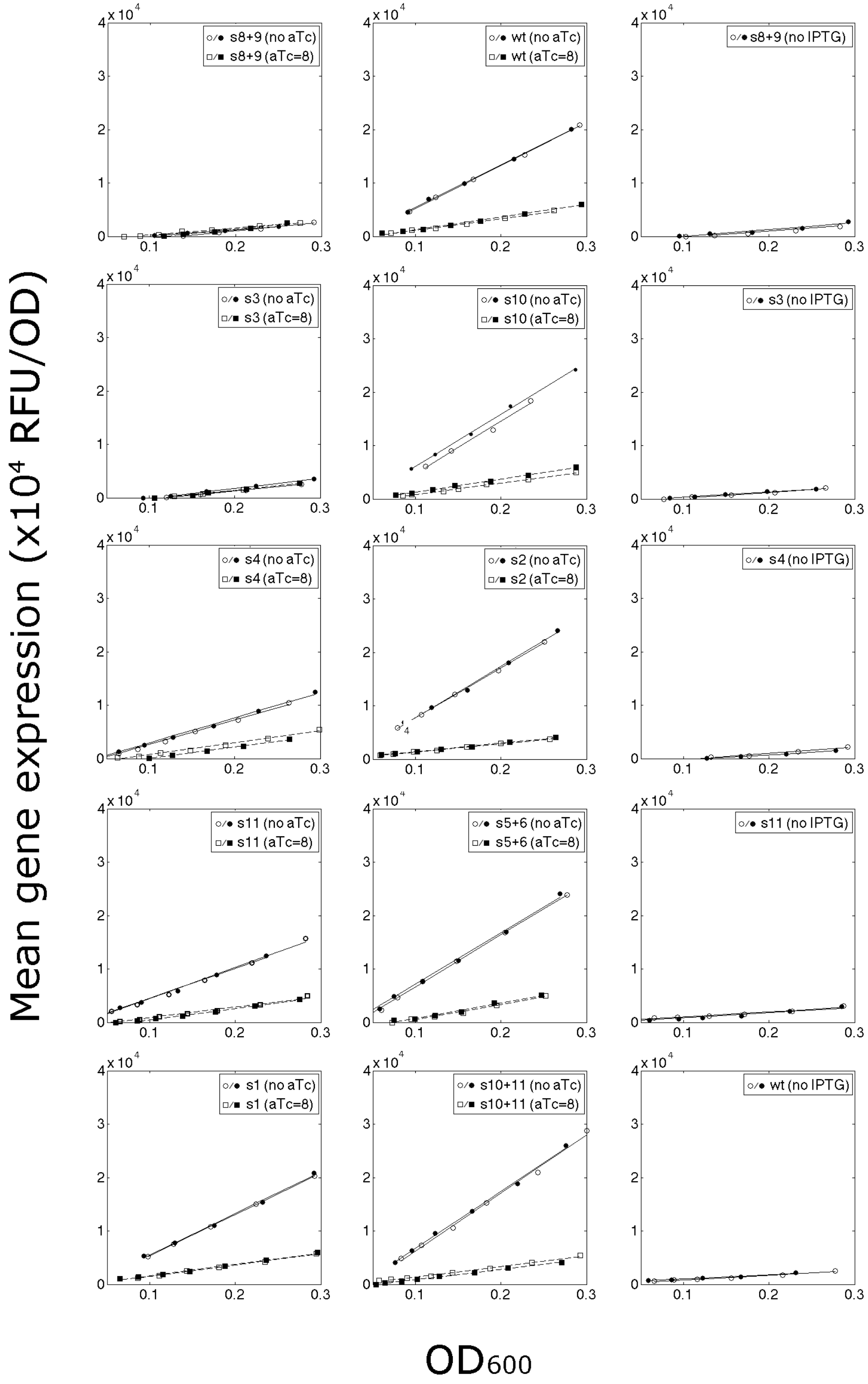

Supplementary Figure S3. **Example for Raw Data for sodB-RyhB used to compile Figure 3 of the main text**. GFP fluorescence is plotted against OD_{600} for the wild type sodB or the mutant strains. Circles-no ryhB induction, Squares- with ryhB induction, duplicates are shown. Lines are given by a linear fit, full line- no ryhB, dashed line- with ryhB. The slope of each line was used to define the “mean gene expression (in RFU/OD unit)”.

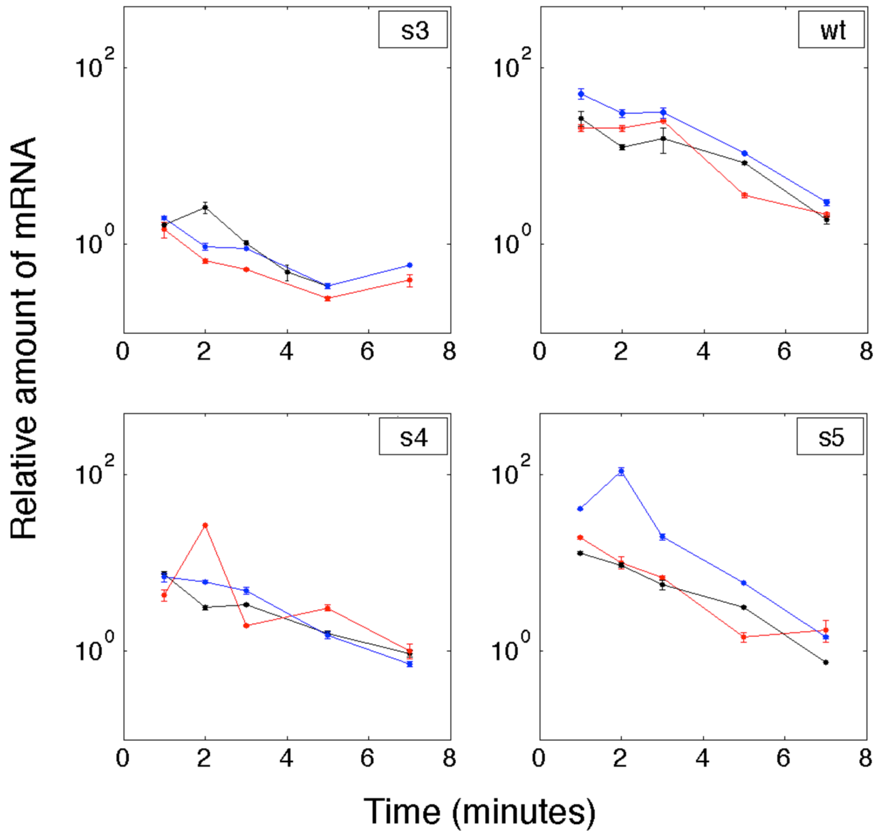

Supplementary Figure S4. **Raw Data used to compile Figure 4A of the main text.** Relative RNA abundance for four different sodB variants at different times after Rifampicin was added, as determined by RT-PCR. Three repeats are shown for each strain with error bars over duplicates for each repeat.

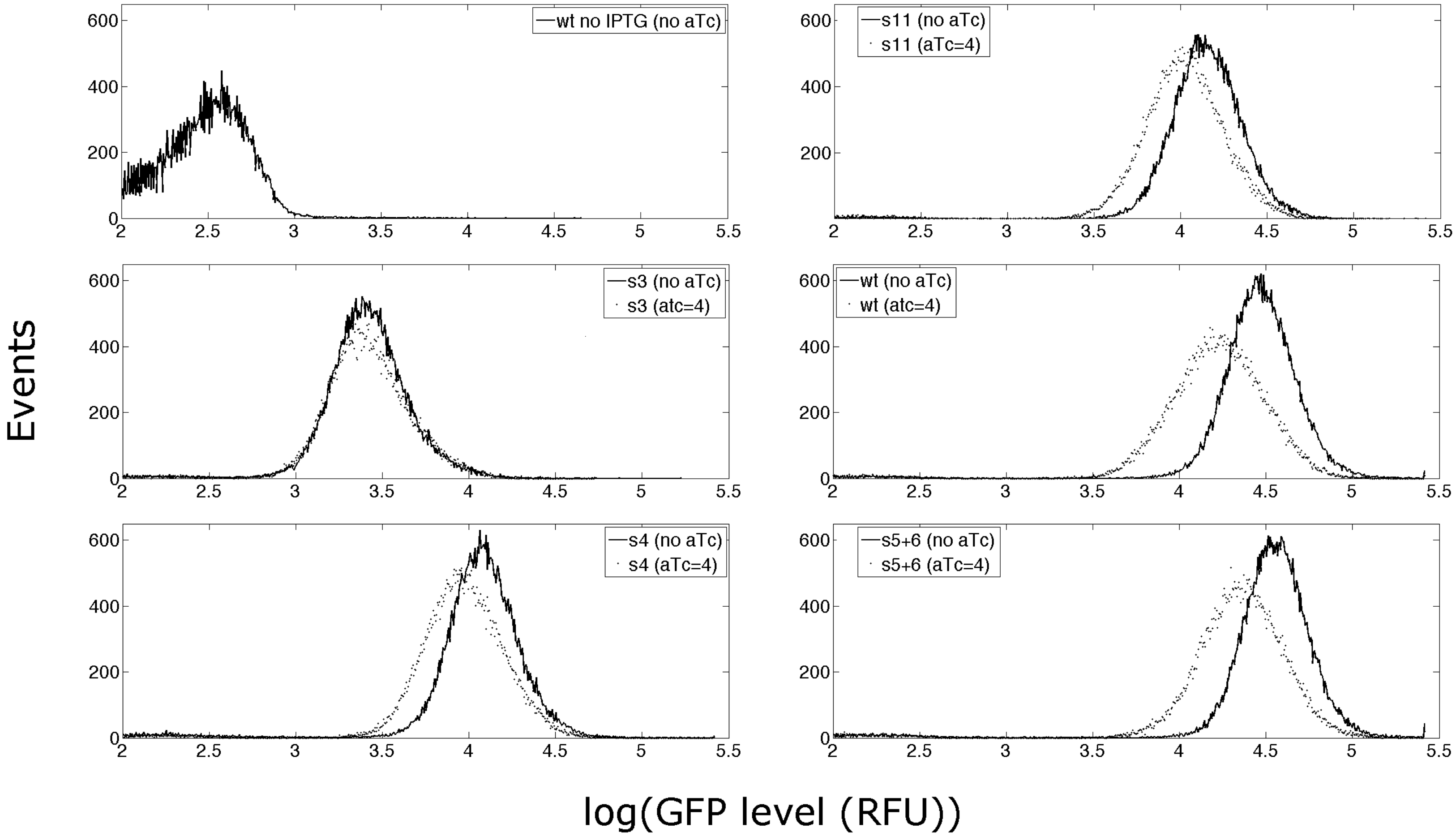

Supplementary Figure S5. **Example for Raw Data used to compile Figure 5 of the main text.** Number of events is plotted against log10(GFP fluorescence (RFU)) for the wild type *sodB* or the mutant strains s3, s4, s5+6 and s11. Full line- no ryhB induction (aTc=0ng/ml), dashed line- with *ryhB* induction (aTc=4ng/ml).

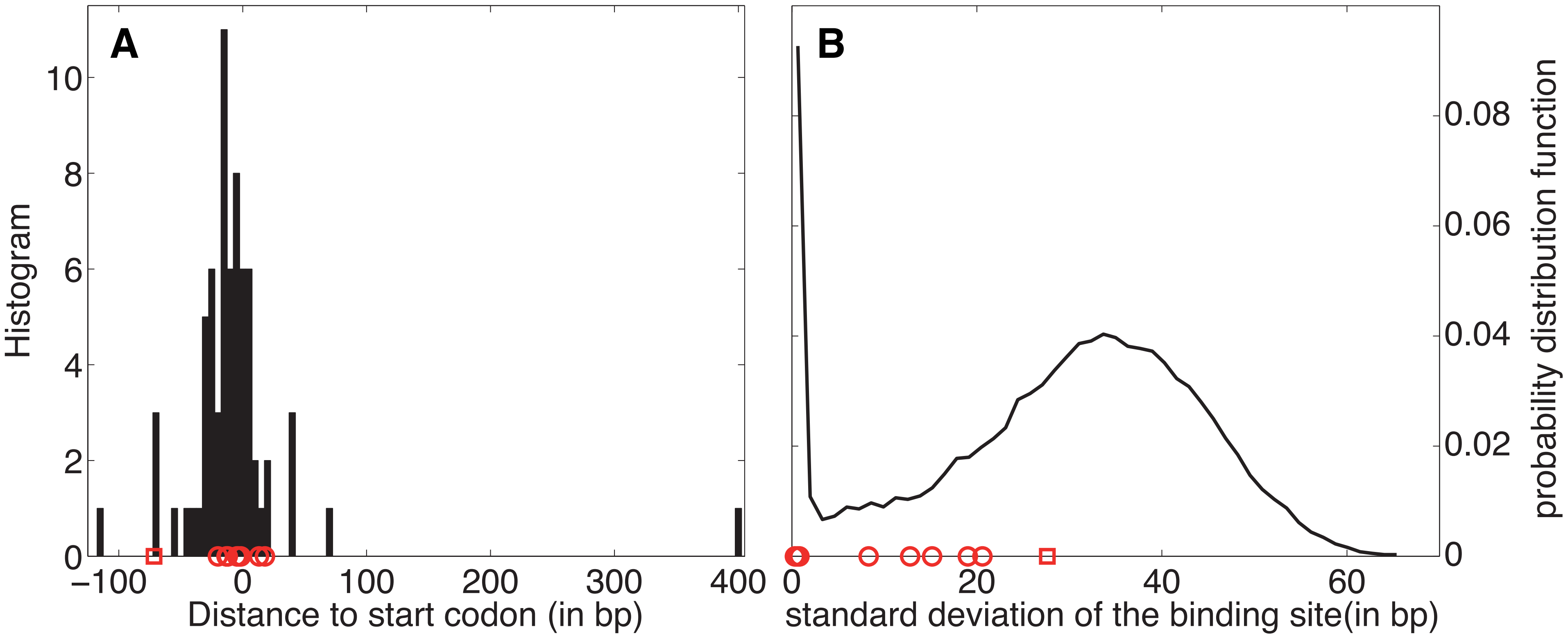

Supplementary Figure S6. **Location and conservation of the position of experimentally known sRNA-mRNA binding sites. (A)** Histograms of the position of binding sites relative to the start codon for about 70 experimentally known repressive bacterial sRNA-mRNA pairs (taken from the sRNA TarBase (25)). **(B)** Probability distribution function of the standard deviation of the positions of the binding sites across Enterobacteriaceae for thousands of “mock” pairs (black line) and 9 real pairs (red circles). Red square corresponds to the binding site of RyhB in *fur*.

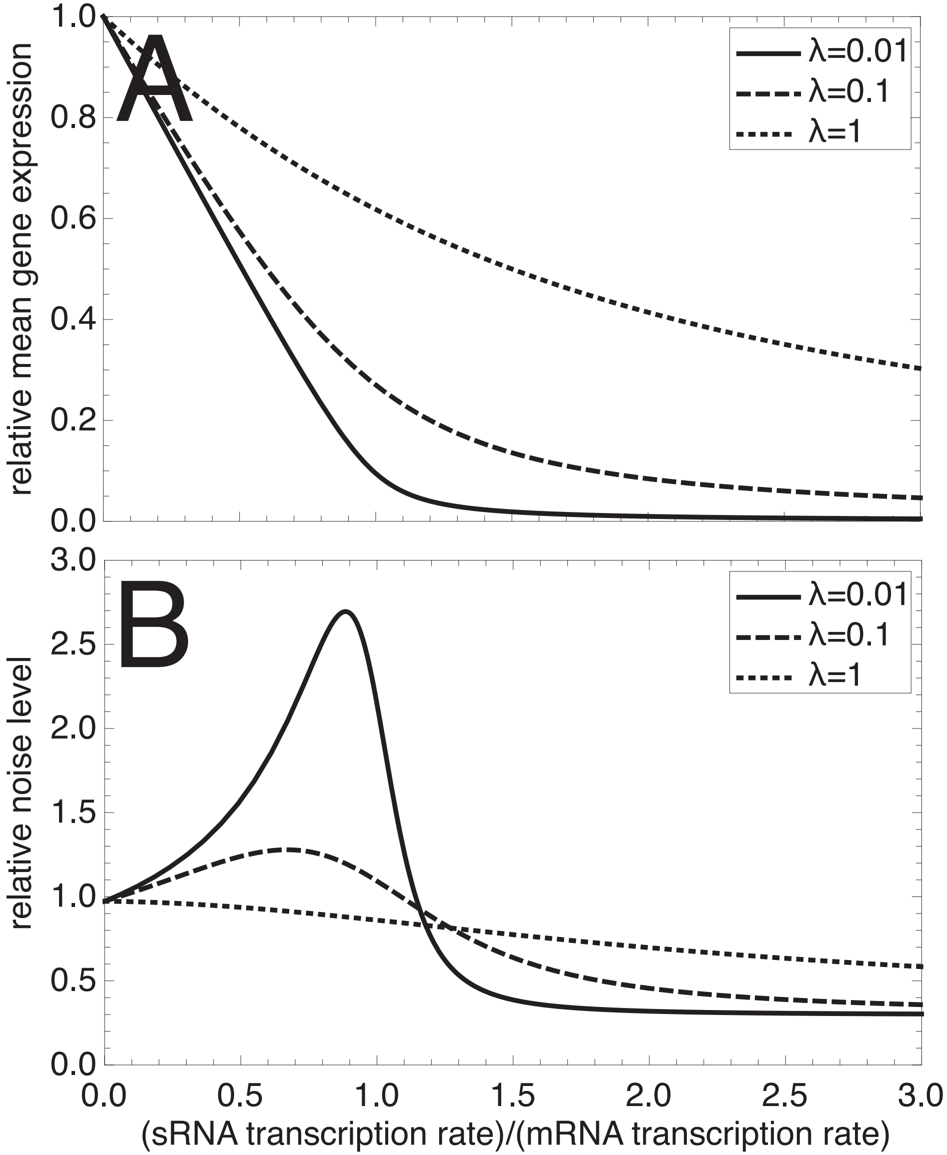

<u>Supplementary Figure S7.</u> **Theoretical predictions for the gene expression level and noise**. **(A)** Relative mean gene expression (defined as the ratio between the mean protein level in the presence and in the absence of sRNA) as a function of the ratio (α_s/α_m) between the sRNA and the mRNA transcription rates, for different values of the leakage rate λ. **(B)** Relative noise level v (defined as the ratio between the intrinsic noise η in the presence of sRNA and the corresponding noise at the same mean protein level in the absence of sRNA) as a function of (α_s/α_m). v <1 means that sRNA regulation is less noisy than transcriptional regulation and v >1 means that sRNA regulation is more noisy. Same legend as in (A). Fixed parameters are (in min^{-1}) $\gamma=1$, $\alpha_m=1$, $\beta_s=0.1$, $\beta_m=0.42$ and $\beta_p=1/60$.

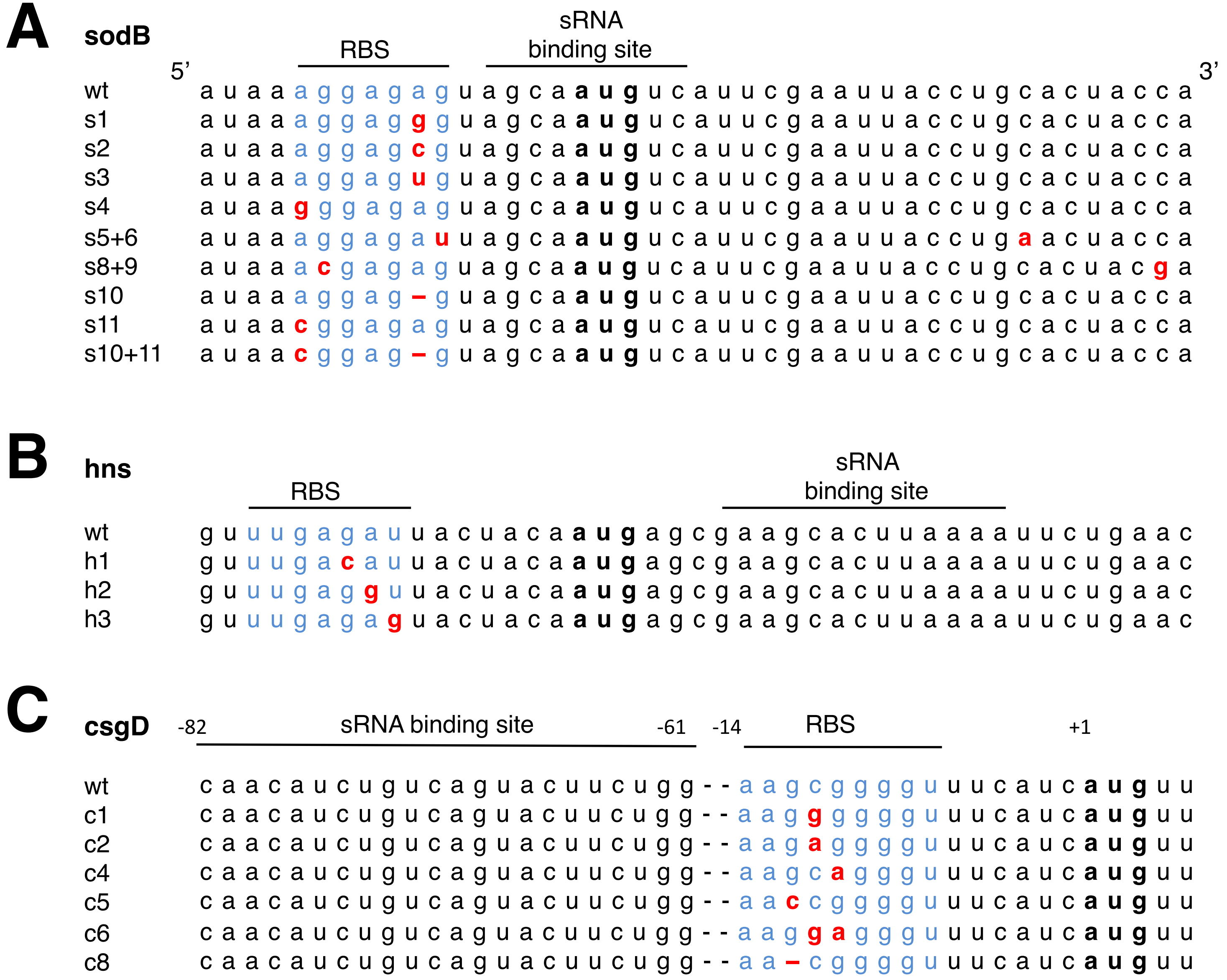

<u>Supplementary Figure S8</u>. 5' Sequences of (**A**) *sodB*, (**B**) *hns* and (**C**) *csgD* variants used in this study. Mutations and deletions in red, RBS in blue and the start codon in bold.

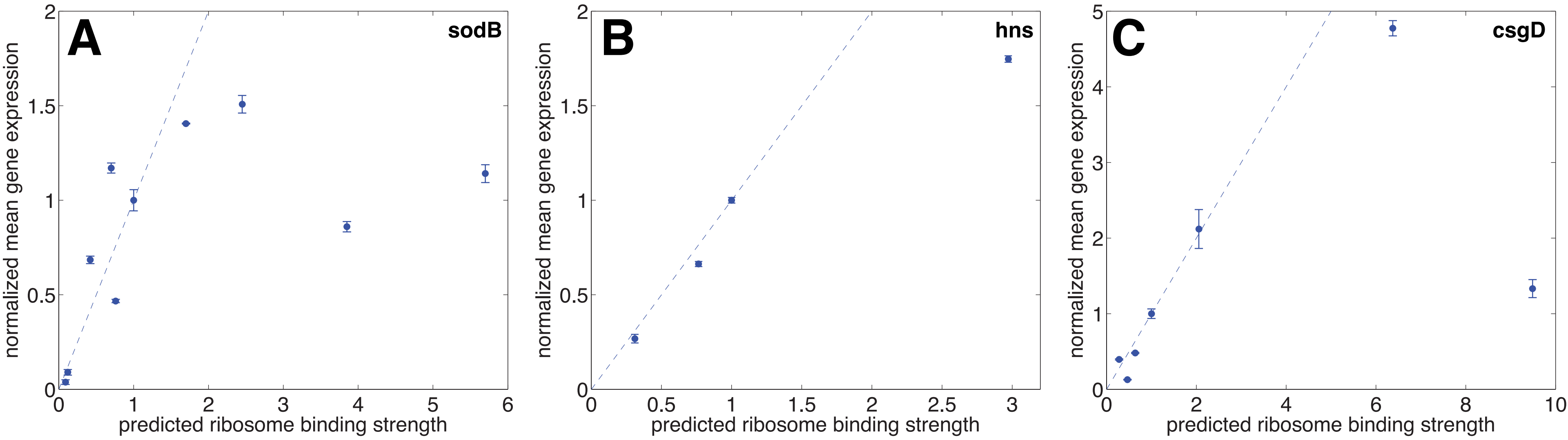

Supplementary Figure S9. **Experimental data correlates with the Ribosome Binding strength prediction**. Correlation between experimental measurements of the mean gene expression of (**A**) *sodB*, (**B**) *hns* and (**C**) *csgD* strain normalized by the expression of the wild type strain and the ribosome binding strength relative to wild type, predicted using the RBS Calculator (31). For sodB, the two are highly correlated at low translation rate (Spearman's rank correlation: R=0.82, p-value=0.03 for strains with a predicted rate less than 2 times the wild-type rate), but translation efficiency saturates at lower levels than predicted. For hns and csgD, the correlation is very high (Spearman's rank correlation: R=1, p-value=0.08 for hns and R=0.86, p-value=0.02 for csgD).

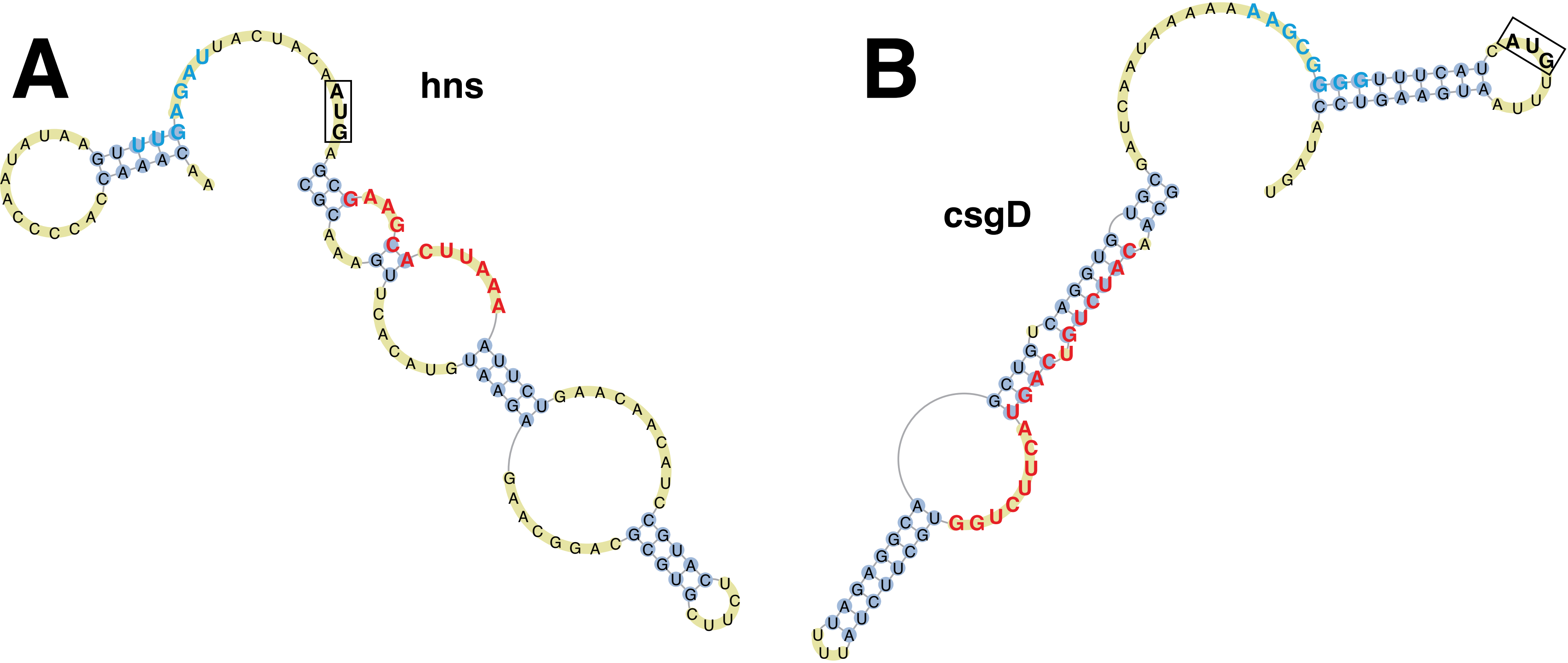

Supplementary Figure S10. **Predicted secondary structure of the 5' UTR of *E. coli* *hns* (A) and *csgD* (B)** (32). Predicted RBS in bold blue, the interaction region with the sRNA in bold red. The start-codon is boxed.

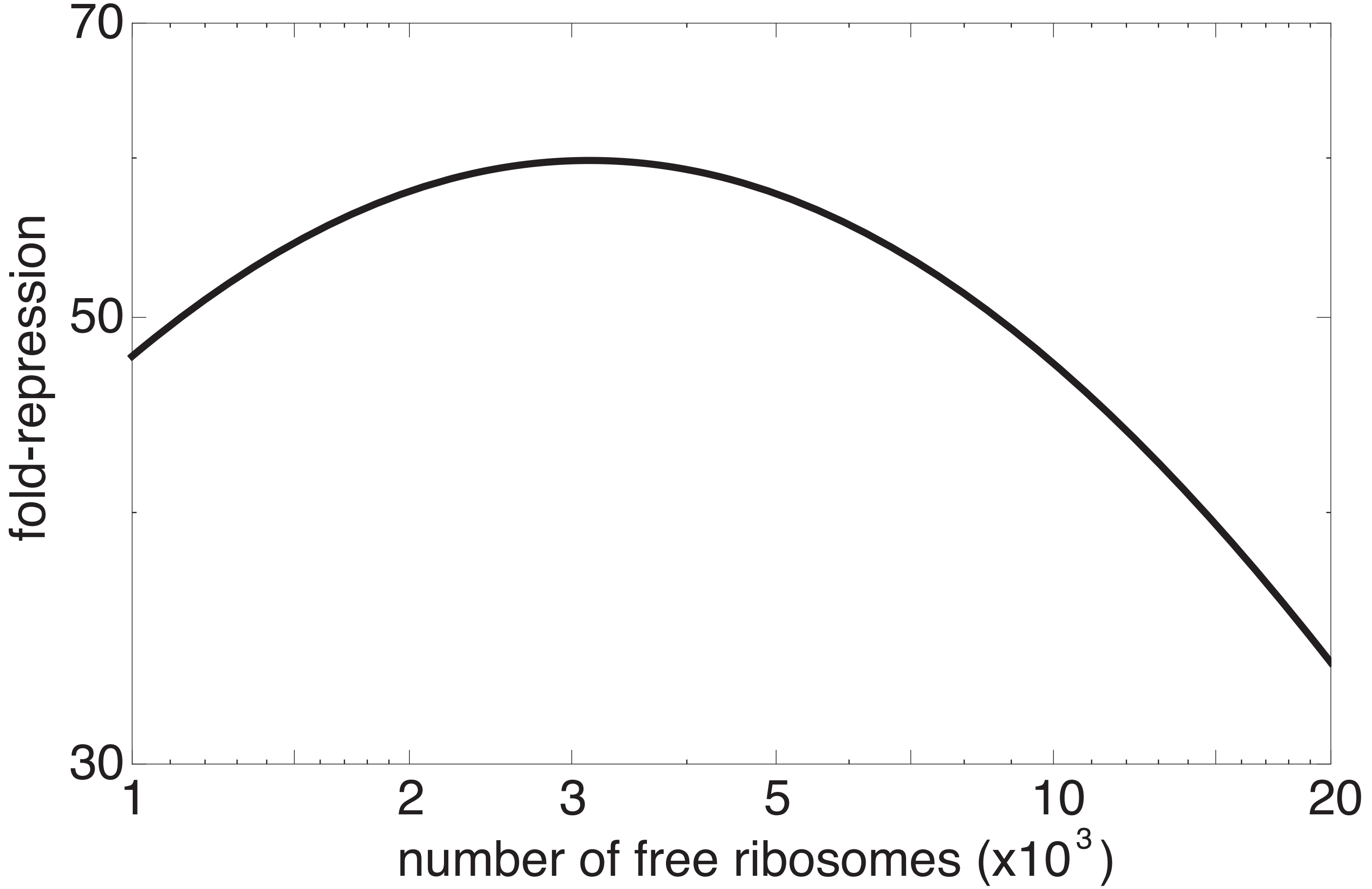

<u>Supplementary Figure S11.</u> **Impact of the number of free ribosomes on the regulation efficiency.** Our model prediction of the fold-repression of the gene expression in the recruitment mode as a function of the number of free ribosomes in the cell. Numbers are typical of exponentially growing cells at 37°C (19). Fixed parameters are (in min^{-1}) $\lambda_0=1$, $\alpha_m=1$, $\beta_s=0.1$, $\beta_{m0}=0.42$, $w=1$, $z=0.001$, $y=100$, $\alpha_s=2$, $K_a=0.001$.

Strain name	Template for site-directed mutagenesis	RBS Sequence	RBS predictor (Salis et al. 2009)	Site-directed mutagenesis primers 5'-3' (reverse primers are the reverse compliment primers, except for with *csgD* variants)
**sodB**		AGGAGAG	1	
S1	pZE12SF	AGGAGGG	3.85	GCAAATTAATAATAAAGGAGGGTAGCAATGTCATTCG
s2	pZE12SF	AGGAGCG	0.70	GCAAATTAATAATAAAGGAGCGTAGCAATGTCATTCG
s3	pZE12SF	AGGAGTG	0.12	GCAAATTAATAATAAAGGAGTGTAGCAATGTCATTCG
s4	pZE12SF	GGGAGAG	0.76	GCAAATTAATAATAAGGGAGAGTAGCAATGTCATTCG
s5+6*	1. pZE12SF<br>2. s5	AGGAGAT	1.7	1. GCAAATTAATAATAAAGGAGATTAGCAATGTCATTCG<br>2. CAATGTCATTCGAATTACCTGAACTACCATATGCTAAAGATGC
s8 +9*	1. pZE12SF<br>2. s8	ACGAGAG	0.09	1. GCAAATTAATAATAAACGAGAGTAGCAATGTCATTCG<br>2. CGAATTACCTGCACTACGATATGCTGGTACCATGA
s10	pZE12SF	AGGAG -G	5.7	CGTATGCAAATTAATAATAAAGGAGGTAGCAATGTCATTCGAATTACC
s11	pZE12SF	CGGAGAG	0.42	AGGCTATTGTACGTATGCAAATTAATAATAACGGAGAGTAGCAATGTC
s10 +11	pZE12SF	CGGAG -G	2.45	AGGCTATTGTACGTATGCAAATTAATAATAACGGAGGTAGCAATGTC
**hns**		UUGAGAU	1	
h1	pAS07	UUGACAU	0.31	CCACCCCAATATAAGTTTGACATTACTACAATGAGCGAAGC
h2	pAS07	UUGAGGU	2.97	CACCCCAATATAAGTTTGAGGTTACTACAATGAGCGAAGCA
h3	pAS07	UUGAGGU	0.76	ACCCCAATATAAGTTTGAGAGTACTACAATGAGCGAAGCAC
**csgD**		AAGCGGGGU	1	
c1	pCsgD::GFP	AAGGGGGGU	4.83	F: ATGTTTAATGAAGTCCATAGTATTCATGG (used for all below)<br>R: GATGAAACCCCCCTTTTTTTATTGATCG
c2	pCsgD::GFP	AAGAGGGGU	1.046	R: GATGAAACCCCTCTTTTTTTATTGATCG
c4	pCsgD::GFP	AAGCAGGGU	0.27	R: GATGAAACCCTGCTTTTTTTATTGATCG
c5	pCsgD::GFP	AACCGGGGU	0.407	R: GATGAAACCCCGGTTTTTTTATTGATCG
c6	pCsgD::GFP	AAGGAGGGU	5.325	R: GATGAAACCCTCCTTTTTTTATTGATCG
c8	pCsgD::GFP	AA-CGGGGU	0.49	R: GATGAAACCCGCTTTTTTTATTGATCG

Supplementary Table S1: Primers used for site-directed mutagenesis to create a library of sodB and hns constructs with different RBS

* A complementary mutation is needed to maintain the secondary structure and the sRNA-binding free energies (Supplementary Figure S2 and Supplementary Table S2). A two-step site-directed mutagenesis was therefore performed, such that the product of the first step is the template for the second one.

Name	Binding Free Energy (kcal/mol)
wt	-9.8
s1	-10.0
s2	-10.2
s3	-8.8
s4	-10.1
s5+6	-8.6
s8+9	-9.0
s10	-11.5
s11	-8.7
s10+11	-10.7

Name	Binding Free Energy (kcal/mol)
wt	-14.3
h1	-14.0
h2	-14.0
h3	-14.2

Supplementary Table S2**: Binding free energies (in kcal/mol) between RyhB and *sodB* mRNA (left), and between DsrA and *hns* mRNA (right), predicted by the bioinformatic software RNAup** (32). For each strain, RNAup was running with the first 110 nucleotides of the mRNA and with the full sRNA sequence. Differences between mutants and wild type are all less than 2 kcal/mol, the typical error bar of the predictions. Between OmrA and *csgD* mRNA, the binding free energy is not affected by mutations in the RBS and is equal to -12.3 kcal/mol.

Supplementary Text:
Quantitative effect of target translation on small RNA efficacy reveals a novel mode of interaction

Anat Lavi-Itzkovitz,[1,2] Neil Peterman,[1,2] Daniel Jost,[1,2,3] and Erel Levine[1]

[1]*Department of Physics and FAS Center for Systems Biology, Harvard University, Cambridge, MA 02138, USA*

[2]*These authors contribute equally to this work*

[3]*Present address: Laboratoire de Physique, École Normale Supérieure de Lyon, CNRS UMR 5672, Lyon, France*

I. MODEL

In this section, we describe in details the three-state model shown in Fig.1B of the main text and in Supplementary figure S1 and how it can be simplified to the system of mass-action equations given in the main text.

A. Full Model

We consider three possible states for the mRNA interaction site: a naked state n, a ribosome-bound state n^* and a small RNA-bound state c. The partial equilibriums between states are described by transition rates (Fig. 1B of the main text, Supplementary figure S1)

$$n + r \underset{k_d}{\overset{k_a}{\rightleftarrows}} n^* \tag{1}$$

$$n + s \underset{k_0^-}{\overset{k_0}{\rightleftarrows}} c \tag{2}$$

$$n^* + s \underset{k_r^-}{\overset{k_r}{\rightleftarrows}} c + r \tag{3}$$

where r stands for ribosomes and s for small RNA molecules. At equilibrium, detailed balance of Eqs.1-3 imposes the relation

$$\frac{k_r}{k_r^-} = \frac{k_d k_0}{k_a k_0^-} = \frac{k_0 K_d}{k_0^-} \tag{4}$$

with $K_d = k_d/k_a$ the dissociation constant of the binding of ribosome to the naked site.

The dynamics of the system is captured by a set of mass-action equations

$$\frac{dn}{dt} = \alpha_m - \beta_{m0} n - k_a r n + k_d n^* - k_0 n s + k_0^- c + \gamma_0 n^* \tag{5}$$

$$\frac{dn^*}{dt} = -\beta_{m0}^* n^* + k_a r n - k_d n^* - k_r n^* s + k_r^- c r - \gamma_0 n^* \tag{6}$$

$$\frac{dc}{dt} = -\beta_c c + k_0 n s - k_0^- c + k_r n^* s - k_r^- c r \tag{7}$$

$$\frac{ds}{dt} = \alpha_s - \beta_s s - k_0 n s + k_0^- c - k_r n^* s + k_r^- c r \tag{8}$$

$$\frac{dp}{dt} = \gamma_0 n^* - \beta_p p \tag{9}$$

$$\frac{dr}{dt} = \alpha_r - \beta_r - k_a n r + k_d n^* - k_r^- c r + k_r s n^* \tag{10}$$

where α_m (α_s) is the mRNA (sRNA) transcription rate, β_{m0}, β_{m0}^*, β_c and β_s are respectively the degradation rate of the naked mRNA, of the ribosome-bound mRNA, of the sRNA-mRNA complex and of the sRNA. Eq.(9) describes the dynamics of the protein encoded by the mRNA with γ_0 its production rate from n^* and β_p its degradation rate. Eq.(10) describes the dynamics of the pool of free ribosomes with α_r the production rate and β_r the degradation rate.

B. Simplified model

To simplify this system, we assume that equilibrium between the naked and the ribosome-bound states is fast and that the concentration r of ribosomes is constant. This leads to $k_a rn = k_d n^*$. Therefore, the dynamics of $m = n + n^*$ the number of sRNA-free mRNAs is given by

$$\frac{dm}{dt} = \alpha_m - \beta_{m0}\left(\frac{1+wx}{1+x}\right)m - \left(\frac{k_0}{1+x} + \frac{k_r x}{1+x}\right)sm + (k_0^- + k_r^- r)c \tag{11}$$

with $x = k_a r/k_d = r/K_d$ the affinity of the naked site with ribosomes ($n = m/(1+x)$ and $n^* = mx/(1+x)$) and $w = \beta^*_{m0}/\beta_{m0}$ the ratio between the degradation rates of the ribosome-bound and the naked states. We go further in the simplification by assuming fast equilibration of the complex, namely $dc/dt \approx 0$ and we eliminate c from previous equations

$$\frac{dm}{dt} = \alpha_m - \beta_{m0}\left(\frac{1+wx}{1+x}\right)m - \left(\frac{\beta_c}{\beta_c + k_0^- + k_r^- r}\right)\left(\frac{k_0}{1+x} + \frac{k_r x}{1+x}\right)sm \tag{12}$$

$$\frac{ds}{dt} = \alpha_s - \beta_s s - \left(\frac{\beta_c}{\beta_c + k_0^- + k_r^- r}\right)\left(\frac{k_0}{1+x} + \frac{k_r x}{1+x}\right)sm \tag{13}$$

$$\frac{dp}{dt} = \gamma_0\left(\frac{x}{1+x}\right)m - \beta_p p \tag{14}$$

We define $y = k_r/k_0$ the ratio of interaction rates of the ribosome-bound and of the naked states with the small RNA and $z = k_0^-/\beta_c$ the ratio between the dissociation rate of the sRNA-mRNA complex and the degradation rate of the complex. Then Eq.4 is equivalent to $k_r^- r = k_0^- xy$ and therefore $(\beta_c/[\beta_c + k_0^- + k_r^- r])(k_0/[1+x] + k_r x/[1+x]) = k_0(1+xy)/[(1+x)(1+z(1+xy))]$ leading to the simplified system of mass-action equations given in the main text:

$$\frac{dm}{dt} = \alpha_m - \beta_m m - ksm \tag{15}$$

$$\frac{ds}{dt} = \alpha_s - \beta_s s - ksm \tag{16}$$

$$\frac{dp}{dt} = \gamma m - \beta_p p \tag{17}$$

with the coarse-grained parameters:

$$\beta_m = \beta_{m_0}\left(\frac{1+wx}{1+x}\right) \tag{18}$$

$$k = k_0\left(\frac{1+xy}{(1+x)(1+z+xyz)}\right) \tag{19}$$

$$\gamma = \gamma_0\left(\frac{x}{1+x}\right) \tag{20}$$

C. Accounting for Hfq in the sRNA-binding parameters

It has to be noted that many parameters used in the full and simplified models are themselves coarse-grained parameters that may account effectively for more microscopic processes. Of particular interest in our study, are the parameters k_0 and k_r that defines the strength of recruitment (via $y = k_r/k_0$). For the RyhB-sodB pair (Geissmann and Touati, EMBO J., 2004) and others (Kawamoto, Koide, Morita and Aiba, Mol. Microbiol., 2006), it has been shown that a RNA-binding chaperone, called Hfq (Brennan and Link, Curr. Opin. Microbiol., 2007), plays a pivotal role in the sRNA regulation. For example in the RyhB-sodB case, Hfq binds strongly to sodB at a site just upstream of the RBS and facilitates the binding of the sRNA (Geissmann and Touati, EMBO J., 2004). In absence of Hfq, sRNA binding remains marginal.

In our model k_0 describes the global interaction between sRNA and naked state. Accounting more precisely for Hfq means that, in addition to Hfq-independent binding of sRNA, k_0 contains an important contribution originating directly from Hfq-related processes. This contribution can be modeled by a two-step process: Hfq binds to the naked

site and then sRNA binds to mRNA:

$$n + h \underset{k_h^-}{\overset{k_h}{\rightleftarrows}} n_h \quad \& \quad n_h + s \underset{k_d^-}{\overset{k_d}{\rightleftarrows}} c + h \tag{21}$$

with n_h the Hfq-bound state. This leads to the following 'microscopic' expression for k_0

$$k_0 \approx k_i + k_d \left(\frac{k_h h}{k_h^-} \right) \tag{22}$$

with k_i (resp. k_d) the Hfq-independent (resp. Hfq-dependent) sRNA binding rate.

Identically, k_r, that describes the global interaction between sRNA and ribosome-bound state, may contain a Hfq-contribution. More specifically, the effect of ribosome on sRNA regulation might work at two levels: (1) it enhances (or represses) the Hfq-independent binding (for example by direct interaction with the sRNA or by allosteric effect on the mRNA secondary structure):

$$n^* + s \underset{k_{r_i}^-}{\overset{k_{r_i}}{\rightleftarrows}} c + r \tag{23}$$

or (2) it enhances (or represses) the binding of Hfq to mRNA:

$$n^* + h \underset{k_{r_h}^-}{\overset{k_{r_h}}{\rightleftarrows}} n_h + r \quad \& \quad n_h + s \underset{k_d^-}{\overset{k_d}{\rightleftarrows}} c + h \tag{24}$$

This leads to the following 'microscopic' expression for k_r

$$k_r \approx k_{r_i} + k_d \left(\frac{k_{r_h} h}{k_{r_h}^- r} \right) \tag{25}$$

Probing and testing the Hfq-dependency on k_0 and k_r would require to perform experiments on Hfq-mutants to vary h or on Hfq-binding site to vary k_h or k_{r_h}.

II. STEADY-STATE SOLUTION

In this section, we compute steady-state properties of the simplified model.

A. Mean values

In absence of small RNA, the average protein level is given by

$$\langle p \rangle_0 = \left(\frac{\gamma_0 \alpha_m}{\beta_p \beta_{m_0}} \right) \left(\frac{x}{1 + wx} \right) \tag{26}$$

As expected, $\langle p \rangle_0$ is an increasing function of the affinity x which saturates for large x-values. The half-life of mRNA is given by

$$\tau_{1/2} \equiv \frac{\log 2}{\beta_m} = \left(\frac{\log 2}{\beta_{m_0}} \right) \left(1 + (1 - w) \frac{x}{1 + wx} \right) = \frac{\log 2}{\beta_{m_0}} + (1 - w) \left(\frac{\beta_p \log 2}{\gamma_0 \alpha_m} \right) \langle p \rangle_0 \tag{27}$$

$\tau_{1/2}$ is therefore a linear function of the mean protein level with a slope proportional to $1 - w$. In general one expects $w \leq 1$, where active translation protects the mRNA from degradation.

In presence of small RNA, putting to zero, the temporal derivatives in Eqs.(15-17) leads to quadratic equations for s and m with unique positive answers:

$$\langle m \rangle = \frac{\alpha_m - \alpha_s - \lambda + \left[(\alpha_m - \alpha_s - \lambda)^2 + 4\lambda\alpha_m \right]^{1/2}}{2\beta_m} \tag{28}$$

$$\langle s \rangle = \frac{\alpha_s - \alpha_m - \lambda + \left[(\alpha_s - \alpha_m - \lambda)^2 + 4\lambda\alpha_s \right]^{1/2}}{2\beta_s} \tag{29}$$

$$\langle p \rangle = \gamma \langle m \rangle / \beta_p = \langle p \rangle_0 \left(\frac{1 - \alpha_s/\alpha_m - \lambda/\alpha_m + [(1 - \alpha_s/\alpha_m - \lambda/\alpha_m)^2 + 4\lambda/\alpha_m]^{1/2}}{2} \right) \tag{30}$$

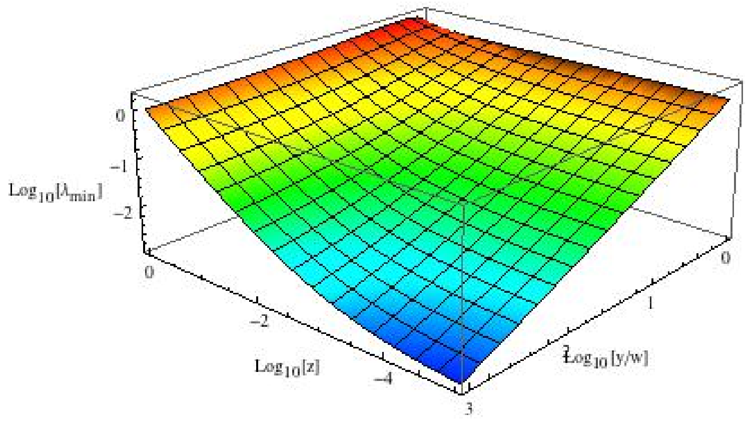

Supplementary figure S 12: Log-plot of the minimal value $\lambda_{\rm min}$ of the leakage rate as a function of z and y/w.

with $\lambda = \beta_s\beta_m/k$ the so-called leakage rate. The efficacy of the regulation is controlled by λ with small values corresponding to efficient silencing. In terms of x, y, w and z, the leakage rate is defined by

$$\lambda = \lambda_0 \frac{(1+wx)(1+z+xyz)}{(1+xy)} \tag{31}$$

with $\lambda_0 = \beta_s\beta_{m0}/k_0$.

The leakage rate λ, and hence the fold-repression at the silenced regime, is strongly dependent on the translation affinity x. If the presence of a ribosome at the interaction site either inhibits sRNA-mRNA interaction or promotes mRNA degradation ($y/w < 1+z$), the leakage rate is a increasing function of x, meaning that sRNA regulation is less efficient for highly translated mRNAs. In contrast, if the presence of a ribosome either promote sRNA-mRNA interaction or protects mRNA from degradation ($y/w > 1+z$), λ is minimal at a positive value for $x_{\rm min} = (\sqrt{(y/w-1)/z}-1)/y$ and the minimum is given by $\lambda_{\rm min} = \lambda_0(w/y)(\sqrt{z(y/w-1)}+1)^2$ (see Supplementary figure S12). High ratio y/w and low z leads to small $\lambda_{\rm min}$ and very efficient regulation. Supplementary figure S13 shows the fold-repression [defined as the ratio between the protein mean levels in absence and in presence of sRNAs, $(\langle p\rangle_0/\langle p\rangle(\alpha_s))$] as a function of the translational activity [defined as $x/(1+wx) \propto \langle p\rangle_0$] for different values of α_s, y, z and w.

To derive the simplified model Eqs.(11-13) we have assumed that the translation of the mRNA does not affect significantly the pool of free ribosomes, ie r is constant ($\approx \alpha_r/\beta_r$). To verify that this assumption does not affect the main conclusions of our work, we compute the outcome of the model if we relax this hypothesis. Supplementary figure S14 shows that the typical behaviors observed in the recruitment ($y/w > 1$) or competition regime ($y/w < 1$) are conserved even if the number of free ribosomes is small and sensitive to high translation. The only visible effect is to slighty decrease the position and the value of maximal sRNA efficacy in the recruitment regime.

B. Fluctuations

Stochastic nature of the underlying biochemical reactions leads to intrinsic fluctuations around the mean signals. Stochastic properties of the mass-action system given in the main text are captured by a master-equation

$$\begin{aligned}\frac{d}{dt}P(m,s,p;t) &= \alpha_m P(m-1,s,p;t) + \beta_m(m+1)P(m+1,s,p;t) + \alpha_s P(m,s-1,p;t) \\ &\quad +\beta_s(s+1)P(m,s+1,p;t) + k(s+1)(m+1)P(m+1,s+1,p;t) + \gamma m P(m,s,p-1;t) \\ &\quad +\beta_p(p+1)P(m,s,p+1;t) - (\alpha_m + \beta_m m + \alpha_s + \beta_s s + ksm + \gamma m + \beta_p p)P(m,s,p;t)\end{aligned} \tag{32}$$

In the limit of weak noise (linear noise approximation), the master equation leads to a set of mass-action equations describing the behavior of the mean values (Eqs.(1a-1c) of the main text) and to a fluctuation-dissipation relation (van Kampen, *Stochastic processes in Physics and Chemistry*, Elsevier)

$$\frac{d}{dt}C = JC + CJ^\dagger + N \tag{33}$$

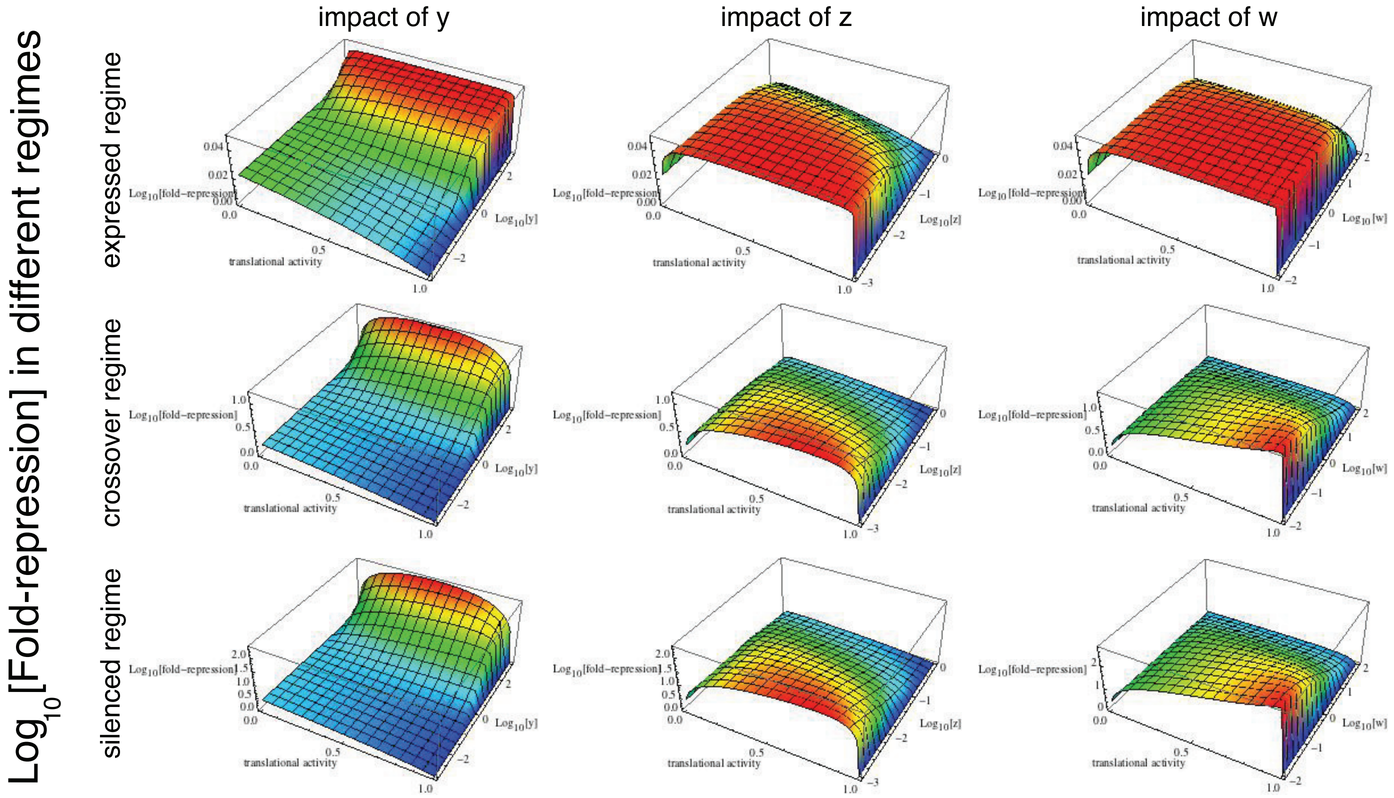

Supplementary figure S 13: Log-plot of the fold-repression as a function of the translational activity and of y (first column), z (second column) and w (third column) in the expressed (first row, $\alpha_s = 0.1\text{min}^{-1}$), cross-over (second row, $\alpha_s = 1\text{min}^{-1}$) and silenced (third row, $\alpha_s = 2\text{min}^{-1}$) regimes. Fixed parameters are $\gamma_0 = 1\text{min}^{-1}$, $\alpha_m = 1\text{min}^{-1}$, $\beta_s = 0.1\text{min}^{-1}$, $\beta_{m0} = 0.42\text{min}^{-1}$, $\beta_p = 1/60\text{min}^{-1}$, $w = 1$, $z = 0.001$ and $y = 100$.

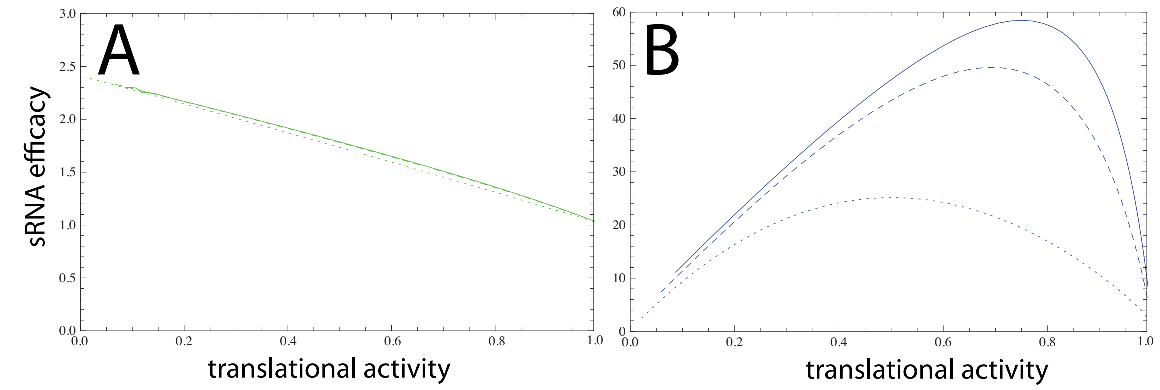

Supplementary figure S 14: sRNA efficacy (fold-repression) as a fonction of the translation activity for different values of α_r (full:10, dashed: 1, dotted: 0.1min^{-1}) in the competition (A,$y = 0.01$) or recruitment (B, $y = 100$) regimes. Fixed parameters are $\gamma_0 = 1\text{min}^{-1}$, $\alpha_m = 1\text{min}^{-1}$, $\beta_s = 0.1\text{min}^{-1}$, $\beta_{m0} = 0.42\text{min}^{-1}$, $\beta_p = \beta_r = 1/60\text{min}^{-1}$, $w = 1$ and $z = 0.001$.

with C the covariance matrix of the system, J the Jacobian of the set of mass-action equations and N the so-called diffusion matrix which integrates the information about the intrinsic noise of each reaction.

In absence of sRNA, the variance of the protein is given by $\sigma_p^2 = C_{p,p} = p(1 + \gamma/(\beta_m + \beta_p)) \approx p(1 + \gamma/\beta_m)$ since $\beta_m \sim \text{min}^{-1} \gg \beta_p \sim \text{h}^{-1}$. Therefore the noise level defined as $\nu = \sigma_p^2/p^2$ equals $(1 + \gamma/\beta_m)/p \equiv (1 + b)/p$ with $b = \gamma/\beta_m$ the so called burst size, ie the average number of protein produced per mRNA. In presence of small RNA, the general analytical expression of σ_p^2 is cumbersome and not really helpful. However in the silenced regime ($\alpha_s \gg \alpha_m$), we find $\nu = (1 + \gamma/(\beta_m + k\langle s\rangle))/p = (1 + b^*)/p$ with $b^* = \gamma/(\beta_m + k\langle s\rangle) \ll b$. In this regime, the noise reduction $((1 + b^*)/(1 + b) \ll 1)$ is significant and is due to the reduction of the effective lifetime of the mRNA

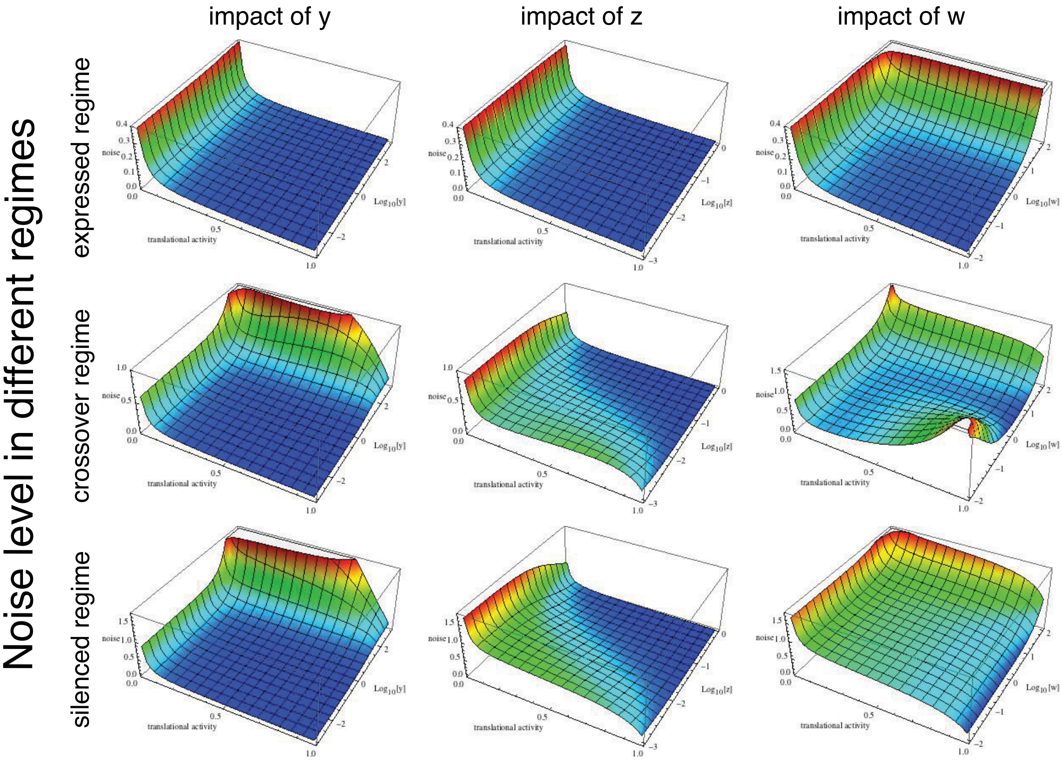

Supplementary figure S 15: Noise level as a function of the translational activity and of y (first column), z (second column) and w (third column) in the expressed (first row, $\alpha_s = 0.1\text{min}^{-1}$), cross-over (second row, $\alpha_s = 1\text{min}^{-1}$) and silenced (third row, $\alpha_s = 2\text{min}^{-1}$) regimes. Fixed parameters are $\gamma_0 = 1\text{min}^{-1}$, $\alpha_m = 1\text{min}^{-1}$, $\beta_s = 0.1\text{min}^{-1}$, $\beta_{m0} = 0.42\text{min}^{-1}$, $\beta_p = 1/60\text{min}^{-1}$, $w = 1$, $z = 0.001$ and $y = 100$.

$(\sim 1/(\beta_m + k\langle s\rangle) \ll 1/\beta_m)$. Supplementary figure S15 shows the noise level for different values of α_s, y, z and w.
